# Evaluation of methods to detect circular RNAs from single-end RNA-sequencing data

**DOI:** 10.1186/s12864-022-08329-7

**Published:** 2022-02-08

**Authors:** Manh Hung Nguyen, Ha-Nam Nguyen, Trung Nghia Vu

**Affiliations:** 1 Information Technology Institute, Vietnam National University in Hanoi, Hanoi, Vietnam; 2 University of Engineering and Technology, Vietnam National University in Hanoi, Hanoi, Vietnam; 3 The Vietnam Institute for Advanced Study in Mathematics, Hanoi, Vietnam; 4 Department of Medical Epidemiology and Biostatistics, Karolinska Institutet, Stockholm, Sweden

## Abstract

**Background:**

Circular RNA (circRNA), a class of RNA molecule with a loop structure, has recently attracted researchers due to its diverse biological functions and potential biomarkers of human diseases. Most of the current circRNA detection methods from RNA-sequencing (RNA-Seq) data utilize the mapping information of paired-end (PE) reads to eliminate false positives. However, much of the practical RNA-Seq data such as cross-linking immunoprecipitation sequencing (CLIP-Seq) data usually contain single-end (SE) reads. It is not clear how well these tools perform on SE RNA-Seq data.

**Results:**

In this study, we present a systematic evaluation of six advanced RNA-based methods and two CLIP-Seq based methods for detecting circRNAs from SE RNA-Seq data. The performances of the methods are rigorously assessed based on precision, sensitivity, F1 score, and true discovery rate. We investigate the impacts of read length, false positive ratio, sequencing depth and PE mapping information on the performances of the methods using simulated SE RNA-Seq simulated datasets. The real datasets used in this study consist of four experimental RNA-Seq datasets with ≥100bp read length and 124 CLIP-Seq samples from 45 studies that contain mostly short-read (≤50bp) RNA-Seq data. The simulation study shows that the sensitivities of most of the methods can be improved by increasing either read length or sequencing depth, and that the levels of false positive rates significantly affect the precision of all methods. Furthermore, the PE mapping information can improve the method’s precision but can not always guarantee the increase of F1 score. Overall, no method is dominant for all SE RNA-Seq data. The RNA-based methods perform better for the long-read datasets but are worse for the short-read datasets. In contrast, the CLIP-Seq based methods outperform the RNA-Seq based methods for all the short-read samples. Combining the results of these methods can significantly improve precision in the CLIP-Seq data.

**Conclusions:**

The results provide a systematic evaluation of circRNA detection methods on SE RNA-Seq data that would facilitate researchers’ strategies in circRNA analysis.

**Supplementary Information:**

The online version contains supplementary material available at (10.1186/s12864-022-08329-7).

## Background

Circular RNA (circRNA) is a type of single-stranded RNA molecule with a closed structure. Unlike the conventional linear RNA, which is a straight nucleotide chain in 5’-3’ direction commonly obtained in transcription, 5’ cap and 3’ poly-A tails do not exist in a circRNA molecule. Instead, a reversed linkage between two ends of the molecule forms its looping structure [1]. This creates some distinctive features in circRNA, for instance, it degrades the effect of exonuclease digestion, thus preserving circular RNAs longer in the cell [2]. CircRNA has also been known to have other diverse functions, including microRNA sponge [3, 4], RNA-binding protein (RBP) sponge [5, 6], gene regulation by competing with pre-mRNA splicing [7], and the parental gene modulation of exon-intron circular RNAs [8, 9]. These typical features attract researchers due to their applications in disease studies, especially as potential biomarkers for cancer diagnosis [10].

The rapid growth of recent studies on circRNA is attributed to the availability of advanced RNA-sequencing technologies that allow to sequence total RNAs including circRNAs [11]. In total RNA sequencing, RNA molecules extracted from cells or tissues are processed to deplete the majority of ribosomal RNAs, usually by using the Ribo-Zero protocol [12]. Then, the remaining RNAs are converted to complementary DNAs to be fragmented and sequenced at one end (single-end mode) or both ends (paired-end mode) of the fragments. Paired-end (PE) reads, which carry more information of a fragment, are usually better than single-end (SE) reads in downstream analysis such as the alignment and detection of genomic rearrangement [13]. However, SE RNA-Seq can economically deliver a larger volume of data, which is suitable for chromatin immunoprecipitation sequencing (ChIP-Seq) or small RNA-Seq. Furthermore, short SE reads, e.g., of 50bp read length, can be used to achieve a robust estimation for the gene-level expression and differential expression analysis to save substantial resources [14, 15]. Multiple SE RNA-Seq datasets are used to study circRNAs in different cell lines of Homo sapiens and Mus musculus [16]. A large amount of SE RNA-Seq data have been generated, which are still a great resource for the community today.

Although the cost of PE RNA-Seq has significantly decreased recently, certain RNA-Seq methods allow only SE reads. For example, cross-linking immunoprecipitation sequencing (CLIP-seq) is used to analyze the interactions between RNAs and RNA-binding proteins (RBPs). CLIP-Seq methods generally start with the cross-linking process of RBPs to RNA-binding sites, followed by the sequencing of binding RNA fragments [17]. Thus, in principle, the data from CLIP-Seq can be used to detect circRNAs. CircRNAs identified from the CLIP-Seq data would be considered evidence for the interactions between circRNAs and RBPs [18]. CLIP-Seq can be used to study the interaction between circRNAs, proteins, and microRNAs, for example, ciRS-7 serves as a binding platform for AGO2 and miR-7 [3], suggesting the regulatory functions of circRNAs [16]. Most CLIP-Seq protocols produce very short SE reads [18], making the process of detecting circRNAs highly challenging.

The key challenge to the methods to detect circRNAs from RNA-seq data is identifying genuine back-splicing junction (BSJ) reads, which cover the linkage of the downstream-3’-splice-donor exon attached to the upstream-5’-splice-acceptor exon of a circRNA. The identification of a BSJ from RNA-Seq data does not always assure a true circRNA. Indeed, exon repetition, genomic tandem duplication, trans-splicing, and reverse-transcriptase template-switching [19] can generate linear transcripts with the duplication of exons forming false positive (FP) BSJs. For convenience, from this point on, we call these transcripts “tandem RNAs”.

A common approach to identify FP BSJs caused by tandem RNAs is utilizing the information of two reads of a pair from the PE RNA-Seq data, following a simple rule: if the paired mate of a supporting-BSJ read is mapped outside the putative region of the circRNA candidate, the reads likely originated from a tandem RNA. However, this rule is not applicable in the SE RNA-Seq situation wherein the mate pair is missing. Theoretically, circRNA detection methods can achieve higher precision in PE RNA-Seq rather than SE RNA-Seq. Read length is another factor that has a great impact on circRNA detection. Some circRNA detection tools such as CIRI2 [20] are reported to be unable to work with RNA-Seq reads shorter than 40bp [18]. To our best knowledge, current comparative studies [21–23] focus on evaluating the performances of circRNA detection methods on PE RNA-Seq data. Furthermore, the impacts of single-end reads, e.g., from the CLIP-Seq, the proportion of false positives from the tandem RNAs, and read lengths pertaining to these methods have not yet been properly investigated.

In this study, we present a systematic evaluation of eight advanced circular RNA detection tools including CIRI2 [20], CIRCexplorer [24], DCC [25], CircRNA_finder [26], Find_circ2 [16], UROBORUS [27], CircScan [28], and Clirc [18] on SE RNA-Seq datasets. In the simulation study, a wide range of read lengths, different proportions of false positives from the tandem RNAs, and sequencing depth are taken into account. Furthermore, we evaluate the impact of the PE mapping information through the difference between the results of the SE datasets and the corresponding PE datasets. Two types of experimental datasets are also used for the evaluation including 1) four SE RNA-Seq datasets from four different cell lines – HEK293, HEK293T, HELA, and HS68; and 2) 124 CLIP-Seq samples from multiple CLIP-Seq protocols. The performances of the circRNA detection methods are assessed using various metrics including sensitivity, precision, F1 score, and true discovery rate.

## Method

### Circular RNA detection tools

Multiple tools and software packages [29] have been developed to detect circRNAs from RNA-Seq data. In general, all such tools require an aligner to identify unmapped reads from the annotated references to discover the BSJs of circRNA candidates. Then, different statistical adjustments and filters such as canonical splicing conditions and minimum supporting reads can be applied to eliminate potential false positives. Even though most of the tools are developed to work on PE RNA-Seq data, a few methods can also be applied to work with SE RNA-Seq data. In this study, we pick methods that can support SE reads for the evaluation. All selected tools use traditional strategies which identify circRNAs through direct BSJ discovery from handling raw sequence reads. Other approaches to predict circRNAs that do not begin with this type of data, such as machine-learning-based methods [29], are not included in the study. In general, we categorize them into two groups: RNA-Seq based methods and CLIP-Seq based methods. The selected RNA-Seq based methods are originally developed to work with RNA-Seq data including CIRI2 [20], CIRCexplorer [24], DCC [25], CircRNA_finder [26], Find_circ2 [16], and UROBORUS [27]. Among these, CIRI2 [20] and CIRCexplorer [24] are the top-performing methods for PE RNA-Seq data according to the most recent comparative studies [21–23]. The CLIP-Seq based methods including Clirc [18] and CircScan [28] are specifically developed for detecting circRNAs from CLIP-Seq data. More details of each method are summarized below.

CIRI2 [20] is one of the most widely used circular RNA detection tools. Using the BWA-MEM [30] alignment result, CIRI2 takes two scanning rounds to find potential BSJs and eliminates false positive circRNAs from tandem forward-splicing junctions (FSJs). In the first round, it evaluates the paired chiastic clipping (PCC) signal of reads to find whether they fit BSJ templates or not. The PCC signal is translated from the CIGAR (Compact Idiosyncratic Gapped Alignment Report) of the read from the BWA alignment. The PE mapping signal is also considered to improve the result; however, this information is not available in the SE data. Further, the condition of the canonical GT/AG splice sites from exon boundaries can be taken into account. In the second round, CIRI2 uses an adapted maximum likelihood estimation based on multiple seed matching for determining the segment location and distinguishing BSJ reads from FSJ reads. This helps handle unbalanced sequences and reduce the false discovery rate.

Find_circ2 [16] performs read alignment using BWA-MEM. Generally, this tool extracts the 20 bp anchors at both sides of the unmapped reads from the first alignment and analyzes their mapping positions to decide whether they stand for plausible circular RNA splicing. It also applies additional steps for specificity improvement such as the GU/AG signal of the splice site and at most two mismatches allowed for a BSJ supporting read. Find_circ2 does not take into account the paired-end mapping information [31].

CIRCexplorer [24] is a circRNA detection tool that can support either TopHat or STAR (the default setting) for alignment. Unmapped reads from the alignment are split and realigned in reverse order to find BSJ reads. An additional alignment to known gene annotation can be performed to adapt sequencing reads’ positions to exon boundaries accurately and consistently.

DCC [25] implements the mapping using STAR aligner and identifies BSJ reads from the set of chimerically aligned reads. DDC applies several filters including the inner-circle region alignment of the mate read in PE sequencing, the existence of GT/AT splicing signal in putative BSJ junctions, and the elimination of candidates mapped to repetitive or homologous regions.

CircRNA_finder [26] is a STAR-based tool. Chimeric junction reads after STAR alignment are filtered by some criteria, which includes matching GT-AG splice sites, at most three mismatches allowed for BSJ supporting reads, and 100kb of maximum distance between the acceptor and donor anchors. For PE RNA-Seq data, it requires the mate read of a BSJ supporting read to stay inside the putative circRNA region.

UROBORUS [27] utilizes TopHat [32] together with Bowtie [33] for alignment to obtain unmapped reads. Then the tool trims the unmapped reads to extract 20bp from two ends (head and tail) of the reads to form artificial paired-end reads. Next, these short paired-end reads are mapped again to the genome using Tophat to get the BSJ reads aligned to the joining region of two back-spliced exons with 1) minimum 20bp of overhang at any of the reads (balanced mapped junctions - BMJ) and 2) less than 20bp of overhang at one end of the reads (unbalanced mapped junctions - UMJ). Finally, some filters are applied, for example, chromosome locaction, maximum gene distance and the minimum number of supporting reads, and etc.

Both Clirc [18] and circScan [28] are CLIP-Seq based methods developed to address the typical issues concerning most CLIP-Seq samples: SE reads with very short read length (<50 bases). Clirc starts with building the sequences of linear transcripts and linearized circRNAs and then maps input RNA reads into the prebuilt annotation using Gsnap [34]. Only reads mapped across the BSJs of linearized circRNAs are input into a filter step. This step takes into account the length of overhangs from both sides of the BSJs (minimum five bases) and the mismatch rates in the overhangs (maximum 0.15).

Using another approach, CircScan maps input RNA data to both genome and transcriptome sequences using Bowtie [35]. Next, the unmapped reads are searched to see if they contain both the donors and acceptors constructed from known transcript annotations to collect BSJ candidates. At this step, the mismatch penalty is considered to score the junction reads. For the samples from PAR-Clip [36], a common type of CLIP-Seq, the method compensates for the mismatches caused by “T to C” mutations at the site of cross-linking of photo-reactive nucleoside 4 4-thiouridine. Then, the BSJs from high-score junction reads are mapped to multiple locations, and the junctions non-overlapping with the aligned reads are excluded. Finally, it shuffles the junction sequences 1000 times to build a null distribution for scoring the BSJs and keeps the significant BSJs with a false discovery rate <5*%*.

### Datasets

#### Simulated datasets

We take into account circRNAs, tandem RNAs, and linear RNAs in the simulation for evaluating circRNA detection methods. The FP BSJs in the simulated datasets are controlled by the ratio between the numbers of tandem RNAs and circRNAs to investigate the robustness of the methods. We consider four ratio settings: 5-95, 30-70, 50-50, 80-20, with the proportion of FP BSJs ranging from low to high. For each FP ratio setting, we simulate both SE RNA-Seq data and PE RNA-Seq data using eight different read lengths including short reads (25, 42, and 50bp), long reads (75, 100, and 150bp), and very long reads (200 and 250bp) to investigate their impact on the performances of circRNA detection tools. The short reads of 25 and 42bp indicate the minimum and median read lengths across samples of the CLIP-seq dataset, respectively.

The simulated data are created by Circall-simulator (https://github.com/datngu/Circall), a simulation tool allowing to simulate RNA-Seq data for both circular RNAs and tandem RNAs [37]. From 11,165 exonic circular RNAs of the HELA cell-line collected from the circBase database [38], we divide them into two sets of tandem RNAs and circRNAs in a specific ratio. We run Salmon [39] on the experimental HELA RNase R- dataset in Table S1 to obtain the expression of linear transcripts. Then, we randomly assign the expression from the linear transcripts to the circRNAs and tandem RNAs for simulation. The simulator utilizes the information of reference annotations and genome sequences to build the pseudo-sequences of the circRNAs and tandem RNAs, Figure S1. Finally, the circRNAs, tandem RNAs, and linear RNAs with their corresponding expression are simulated using Polyester [40] to generate a simulated paired-end RNA-Seq dataset. We concatenate two reads of a paired-end sample to build the corresponding single-end RNA-Seq dataset.

#### Experimental RNA-Seq datasets

Table S1 summarizes four experimental PE RNA-Seq datasets of human cell lines: HEK293, HELA, HS68, and HEK293T. All datasets contain 1) a total RNA sample (RNase R-) and 2) a total RNA sample with RNase R treatment (RNase R+). The RNase R+ samples are not supposed to have linear RNAs; thus, they can be used for validating the circRNAs detected from the RNase R- samples. Multiple samples of a dataset are merged to build a single PE sample, and two reads of the PE sample are combined to produce an SE RNA-Seq sample for evaluation.

Briefly, a RiboMinus kit is used for the depletion of ribosomal RNA in all samples across four datasets. Then the libraries are prepared using TruSeq protocol and sequenced by Illumina HiSeq platforms. The HEK293 dataset [41] has an RNase R- sample (SRR3479243) and RNase R+ sample (SRR3479244) with the PE reads of 150bp length. The HELA dataset [42] contains four samples equally divided into two groups: two original samples (SRR1637089 and SRR1637090) and two equivalent samples after RNase R treatment (SRR1636985 and SRR1636986) of 101bp PE reads. The HS68 dataset [43] includes two samples SRR444975 and SRR445016 for data without and with RNase R treatment, both containing 100bp PE reads. The HEK293T dataset [44] consists of four RNase R- samples (SRR1562287, SRR1567913, SRR1567914, and SRR1567915) and two RNase R+ samples (SRR2048277 and SRR2048278) of 100bp PE reads. The details of the datasets are referred to the original studies.

#### CLIP-Seq datasets

We collect a list of 124 public samples of high throughput cross-linking immunoprecipitation sequencing (CLIP-Seq) datasets from three cell lines, HELA (N = 63), HEK293 (N = 42), and HEK293T (N = 19), for evaluation, Table S2. These datasets are originally from 45 different studies providing the RNA sequencing of RNAs linked to 46 different RBPs using various CLIP-Seq protocols including HITS-CLIP, iCLIP, irCLIP, miCLIP, PAR-CLIP, PAR-CLIP-MeRIP, and PAR-iCLIP. A majority of the samples (80%) have short reads (≤50bp) with a median of 42bp. Details of all CLIP-Seq samples are provided in Table S2 in the supplementary document.

#### Quality control

The fastq files of both the experimental RNA-Seq and CLIP-Seq datasets are subjected to quality control by FastQC (https://www.bioinformatics.babraham.ac.uk/projects/fastqc/). We exclude 16 CLIP-Seq samples which do not pass the quality scores per sequence qualification of FastQC from downstream analysis. The information of quality control for individual samples are provided in Table S2 in the supplementary document.

### Performance metrics and method implementation

Six RNA-Seq based methods including CIRI2 version 2.0.6, CIRCexplorer version 1.1.10, DCC version 0.5.0, CircRNA_finder version 1.2, Find_circ2 version 1.99, and UROBORUS version 2.0.0 are used with all datasets. Two CLIP-Seq based methods Clirc version 0.1.0 and CircScan version 0.1 [28] are only used for the comparison between the simulated SE datasets and the CLIP-Seq datasets. Sequence reads of all datasets are mapped to the reference genome and transcriptome of UCSC hg19 Homo sapiens. We set two as the minimum supporting reads in all methods. Further, other parameters are used with the methods’ default settings.

In the simulation study, the results of each tool are collated with the true circRNAs from the simulation setting to calculate sensitivity, precision, and F1 score for comparison.

1 $$ \begin{aligned} \text{Sensitivity} = \frac{\text{Number of the discovered true positives} }{\text{The total number of true circRNAs}}, \end{aligned}  $$

2 $$ \begin{aligned} & \text{Precision} =\\ &\frac{\text{Number of the discovered true positives} }{\text{The total number of circRNAs discovered through the method}}. \end{aligned}  $$

3 $$ \text{F1} = 2\times\frac{\text{Precision} \times \text{Sensitivity}}{\text{Precision} + \text{Sensitivity}}.  $$

For the RNA-Seq data (RNase R-), we follow the common approach [21] that uses the corresponding RNase R treated sample (RNase R+) for the evaluation. First, the expression of the circRNA candidates of a sample is normalized by the sample’s library size. A circRNA from the RNase R- sample is considered “non-depleted” if its expression is less than or equal to its expression in the corresponding RNase R+ sample. For each method, We rank the detected circRNAs by their supporting reads and use the non-depleted circRNAs to compute the true discovery rate for method comparison.

Since both the CLIP-Seq based methods CircScan and Clirc are developed for short-read CLIP-Seq data, not long-read RNA-Seq data, we do not use the non-depleted circRNAs for the assessment in the CLIP-Seq datasets. For a fair comparison, we build a set of “true positives” for each cell line from the circRNAs, which are discovered from the RNase R+ samples by at least two methods. Then, all the methods are evaluated based on these "true positive" sets.

## Results

### Simulated datasets

#### Performances of the circRNA detection methods across read lengths

**Fig. 1:**
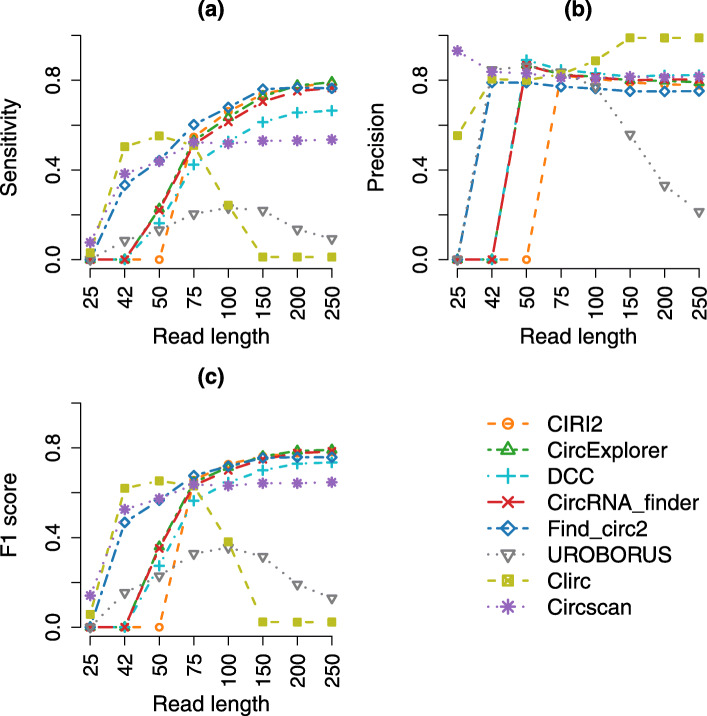
Performances of all circRNA detection methods with the simulated SE datasets with the ratio setting 30-70 across read lengths. The x-axis presents the read length of the simulated samples. The y-axis in panels (a), (b), and (c) presents sensitivity, precision, and F1 score respectively

Figure 1 presents the performances of the methods in the ratio setting of 30-70 (30% FP BSJs for tandem RNAs and 70% TP BSJs for circRNAs). The sensitivities of most methods except that of Clirc and UROBORUS are positively correlated with the read length of the data. The sensitivity of Clirc increases from a read length of 25bp to 50bp and then decreases from 75bp down to nearly zero at 150-250bp, whereas the result of UROBORUS follows a bell-curved shape with the peak at 100bp. The trend is similar for the other FP ratio settings, see Figures S2-4.

CLIP-Seq based methods and Find_circ2 perform better than the rest of the methods with the short-read datasets. None of the RNA-Seq based methods reports circRNAs for the read length of 25bp, and among the methods, only Find_circ2 and UROBORUS discovers circRNAs from the data with 42bp read length. For the shortest read length (25bp), CircScan outperforms Clirc in sensitivity, precision, and F1 score across all FP ratio settings. However, Clirc performs better in terms of both sensitivity and F1 score than CircScan in other cases of the short reads (42bp and 50bp); see Fig. 1 and Figures S2-4.

For the longer-read datasets, UROBORUS and Clirc achieve the worst and best precision respectively. The precision of other methods is not significantly different, making their F1 scores across read lengths highly dependent on their sensitivity. Thus, for those methods, increasing read length generally improves the F1 score. The performances of CIRI2, CircExplorer, and Find_circ2 are comparable in most FP ratio settings. The details of their performances with the simulated datasets are provided in Table S3-6 in the supplementary document.

#### Impact of tandem RNAs on the performances of the circRNA detection methods

**Fig. 2:**
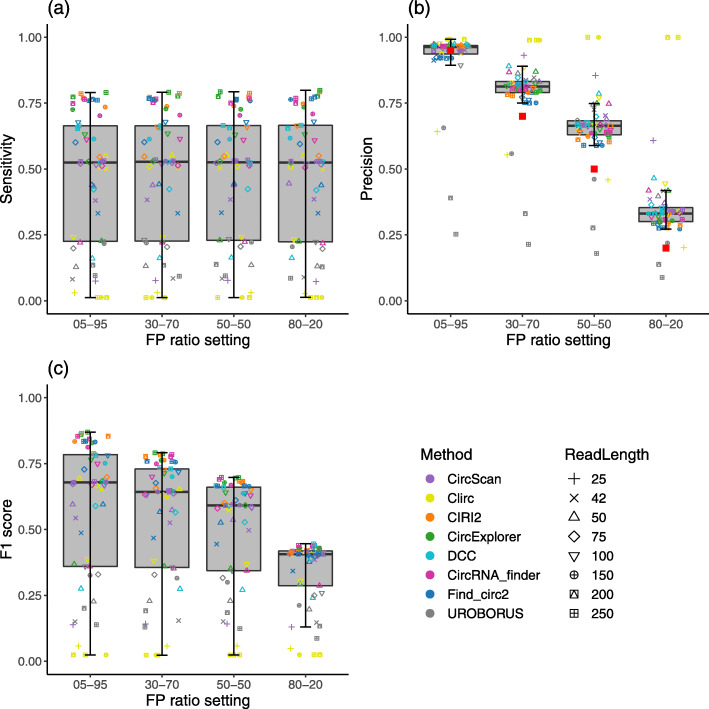
Performances of all circRNA detection methods with the simulated SE datasets across FP ratio settings. A box plot presents the results from all simulated SE datasets of a setting across all methods and read lengths. Panels (a), (b), and (c) presents the results for sensitivity, precision, and F1 score respectively

We assess the robustness of circRNA detection methods with regard to the FP BSJs using four FP ratio settings covering different proportions of tandem RNAs from low to high. Figure 2(a) shows that the methods’ sensitivity is nearly unchanged regardless of the increase in the ratio of the FP BSJs. In contrast to sensitivity, the methods’ precision has a strong negative correlation with the proportion of tandem RNAs, as shown in Fig. 2(b). The precision median values are higher than the true positive rates in the ratio settings (red squares). In other words, these methods are better than the naïve approach that reports all BSJs. However, the substantial decrease in precision in the ratio settings with high FP rates indicates that the methods are severely affected by the tandem RNAs. Since the sensitivities are extremely close across the ratio settings, the methods’ F1 scores are also negatively correlated with the proportion of the FP BSJs, as indicated in Fig. 2(c).

#### Impact of sequencing depth on the performances of the circRNA detection methods

**Fig. 3:**
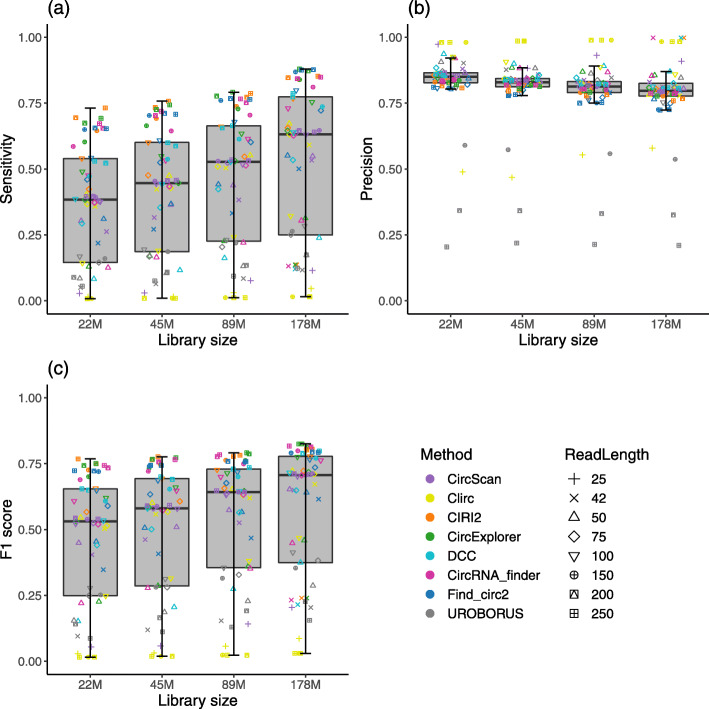
Performances of all circRNA detection methods with the simulated SE datasets across library sizes. A box plot presents the results from all simulated SE datasets of a setting across all methods and read lengths. Panels (a), (b), and (c) presents the results for sensitivity, precision, and F1 score respectively

To investigate the impact of sequencing depth, we simulate more data with different library sizes for all simulated SE datasets with the ratio setting 30-70. Particularly, for each dataset with a library size of *L*=89M reads, we use the same setting to generate three extra simulated datasets with the fold-changes of 0.25, 0.5, and 2 between their library sizes and *L*, resulting 22M, 45M, and 178M reads, respectively. We perform all methods using the same parameters for these SE simulated datasets. The results of all methods are summarized in Fig. 3. As expected, the increase of sequencing depth allows the circRNA detection methods to identify more BSJs, therefore, can significantly improve the methods’ sensitivity, Fig. 3(a). However, a larger library size also produces more FP BSJs, which makes a slight decrease in the methods’ precision for these simulated datasets, Fig. 3(b). Overall, similar to the sensitivity, the F1 scores of the methods are highly correlated with the sequencing depth, Fig. 3(c).

#### Performance differences between single-end reads and paired-end reads

We further evaluate the impact of the PE mapping information by comparing the results of the simulated SE data with the output of corresponding simulated PE datasets. CircScan and Clirc can run only on SE RNA-Seq data, so they are excluded from any PE data analysis. For each simulated PE dataset, we compute the precision difference of a method from the corresponding simulated SE dataset. Thus, the difference indicates that a method has an improvement in precision for the simulated PE data. The calculation is done to all datasets across read lengths and FP ratio settings, as depicted in Figures S5-8. A similar procedure is performed to obtain the sensitivity difference (Figures S9-12) and F1 difference (Figures S13-16).

Since Find_circ does not take into account the paired-end mapping information [31], its results are almost the same for both simulated SE and PE datasets. Most remaining methods perform better in precision for the simulated PE datasets; see Figures S5-8. The three methods CircExplorer, DDC, and CircRNA_finder achieve the most improvement in precision for PE reads at read lengths of 250bp, while CIRI2 has similar precision differences across all read lengths. Longer reads generally further reduce sensitivity for the simulated PE datasets, indicated in Figures S9-12. UROBORUS also obtains an improvement in precision for PE reads at 200 and 250bp, but its precision is worse for shorter PE reads. Furthermore, UROBORUS reports a better sensitivity for PE reads across read lengths. Similar patterns are found with regard to the F1 scores, as shown in Figures S13-16. The F1 differences of CIRI2 are very small (<0.05) but mostly positive, indicating that the performance of this method is slightly better for the PE datasets in general. The F1 differences of UROBORUS have the same trend with larger values. The F1 scores of CircExplorer, DDC, and CircRNA_finder for the PE datasets clearly improve with the read length of 50bp. However, longer reads including 150bp, 200bp, and 250bp degrade their F1 scores. Moreover, the increase of FP rates generally reduces the F1 differences between PE and simulated SE datasets.

### Experimental RNA-Seq datasets

**Fig. 4:**
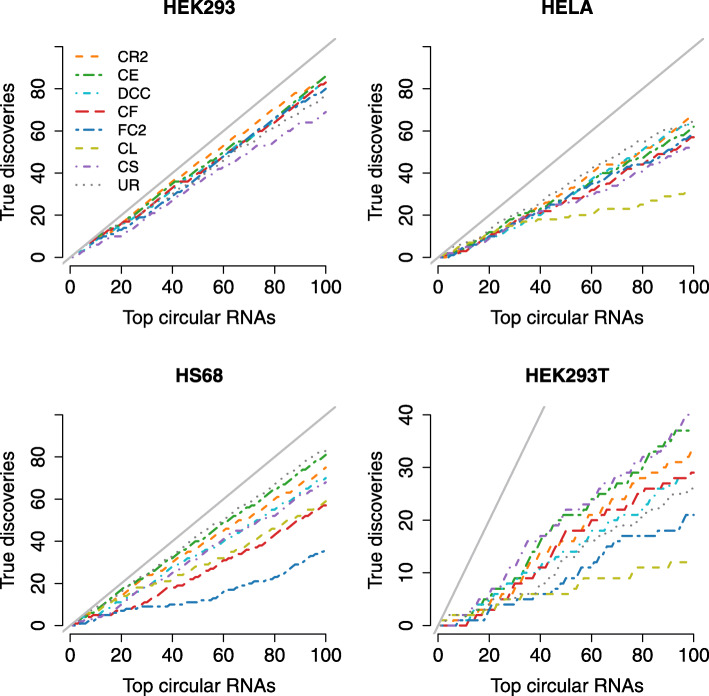
Comparison of all circRNA detection methods in the single-end experimental RNA-Seq datasets using top 100 circRNAs. The x-axis presents the number of top circRNAs ranked by expression. The y-axis indicates the number of true positives (non-depleted circRNAs) in the top circRNAs. The black solid diagonal line presents the perfect true discovery rate

Figure 4 presents the true discovery curves of all methods in the top 100 circRNAs ranked by their expression. The x-axis denotes the number of top circRNAs and the y-axis refers to the number of true positives in the top circRNAs. The methods with their curves closer to the diagonal black line indicating 100% true discovery rate (TDR) are better. The results show that CIRI2 and CIRCexplorer perform rather well and are among the top-performing methods for all datasets. CIRCexplorer has outperformances for the HS68 and HEK293T datasets, while CIRI2 is the best method for the HEK293 datasets. UROBORUS performs rather well for HELA and HS68 datasets, however, it is not among top-performing methods for the other datasets. Further, Find_circ2 and CircRNA_finder deliver the worst performances for the HS68 dataset. Since all experimental RNA-Seq datasets in this study have long reads (≥100bp), Clirc gives very poor performances. The method does not even report any circRNAs in the HEK293 dataset with a read length of 150bp. These results are also concordant with the simulation study. CircScan delivers poor performances with the HELA, HEK293, and HS68 datasets but performs well with the HEK293T dataset. Extending the curves to top 1000 and all circRNAs (Figures S17 and S18), CIRI2 clearly outperforms the methods for HEK293 and HELA datasets and becomes comparable to CircExplorer with HS68 and HEK293T datasets. UROBORUS detects less than 1000 circRNAs across all datasets and its performance drastically reduces for lower-ranked circRNAs. The performances of Find_circ2 are not stable. The method is among the top-performing methods for the HEK293 and HELA datasets but shows the worst performance with the HS68 and HEK293T datasets. Compared with the CLIP-Seq based methods, all RNA-Seq based methods report substantially more circRNAs across all datasets. The total numbers of circRNAs and non-depleted circRNAs detected through individual methods for each dataset is provided in Table 1.

**Table 1:** Results of all circRNA detection methods for single-end and paired-end experimental RNA-Seq datasets across four cell lines. The columns Total, Non-dep, and Percent indicate the total detected circRNAs, the number of non-depleted circRNAs, and the proportion of the non-depleted RNAs respectively. Clirc and CircScan are not applicable to the paired-end datasets

**Dataset**	**Method**	Single-end				Paired-end		
		Total	Non-dep	Percent		Total	Non-dep	Percent
**HEK293**	Ciri2	5841	3413	0.584		4397	3018	0.686
	CircExplorer	4138	2393	0.578		1999	1389	0.695
	DCC	2366	1389	0.587		301	193	0.641
	circRNA_finder	5015	2656	0.530		2244	1510	0.673
	find_circ2	4607	2899	0.629		4605	2897	0.629
	UROBORUS	469	293	0.625		840	453	0.539
	Clirc	0	0	0.000		.	.	.
	CircScan	970	578	0.596		.	.	.
**HELA**	Ciri2	7349	2981	0.406		6205	2885	0.465
	CircExplorer	5469	2010	0.368		2728	1362	0.499
	DCC	3704	1225	0.331		452	215	0.476
	circRNA_finder	7086	2255	0.318		3299	1520	0.461
	find_circ2	7529	2785	0.370		7520	2786	0.370
	UROBORUS	640	266	0.416		1469	473	0.322
	Clirc	136	37	0.272		.	.	.
	CircScan	2269	943	0.416		.	.	.
**HS68**	Ciri2	5331	3406	0.639		4818	3198	0.664
	CircExplorer	3950	2533	0.641		2683	1847	0.688
	DCC	3122	1492	0.478		1747	1066	0.610
	circRNA_finder	6402	2806	0.438		3520	2021	0.574
	find_circ2	9070	3395	0.374		9076	3404	0.375
	UROBORUS	551	356	0.646		1138	653	0.574
	Clirc	114	68	0.596		.	.	.
	CircScan	1727	1138	0.659		.	.	.
**HEK293T**	Ciri2	8486	1806	0.213		7719	1435	0.186
	CircExplorer	5347	1339	0.250		4102	679	0.166
	DCC	3896	750	0.193		2548	376	0.148
	circRNA_finder	8237	1521	0.185		4924	751	0.153
	find_circ2	11891	1783	0.150		11896	1783	0.150
	UROBORUS	794	157	0.197		1545	351	0.227
	Clirc	166	23	0.139		.	.	.
	CircScan	2080	648	0.312		.	.	.

Most methods achieve substantially fewer circRNAs with the PE RNA-Seq datasets; see Table 1. The number of circRNAs detected by DCC with the PE mode decreases up to 80% in the HEK293 and HELA cell lines. In contrast, the number increases about two folds for the PE mode of UROBORUS across all datasets. Similar to the simulation study, the results of Find_circ2 with both SE and PE data are nearly the same across cell lines. CIRI2, CircExplorer, DCC, and CircRNA_finder gain better proportions of non-depleted circRNAs from the PE data in HEK293, HELA, and HS68 cell lines but show the worse performances for the PE dataset of HEK293T cell line. We further compare the results of PE and SE datasets based on their differences in true discovery rate (TDR) across methods. The circRNAs of each method are ranked by their expression; then, the TDR is calculated for top circRNAs in both PE and SE datasets. For the PE HELA and HEK293 datasets, all methods, except Find_circ2 and UROBORUS, generally gave better TDR with the PE data for highly expressed circRNAs, as provided in Figures S19 and S20. In contrast, the results of the SE dataset are better than the results of the PE dataset for the highly expressed circRNAs in the HEK293T cell line; see Figure S21. Only DCC and CircRNA_finder achieved better TDR for the highly expressed circRNAs of the PE dataset in the HS68 cell line, as shown in Figure S22.

### CLIP-Seq datasets

**Fig. 5:**
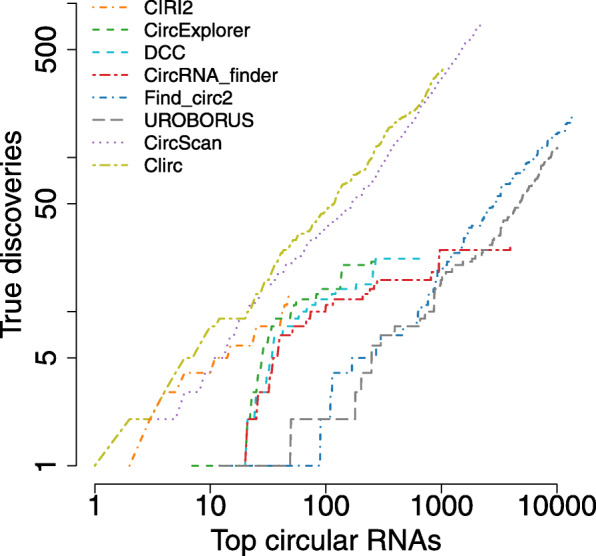
Comparison of all circRNA detection methods with the CLIP-seq dataset using true discovery rate.. The x-axis presents the number of top circRNAs ranked by expression. The y-axis indicates the number of true positives (non-depleted circRNAs) in the top circRNAs. The black solid diagonal line presents the perfect true discovery rate. The x-axis and y-axis are presented in log-scale

From the results of the simulation study, the CLIP-Seq based methods are superior to RNA-Seq based methods with the short-read simulated datasets. In the CLIP-Seq datasets, 80% samples constitute short-read (≤50bp) data (Table S2), making both CircScan and Clirc outperform the other methods, as depicted in Fig. 5. Among RNA-Seq based methods, CIRI2 obtains the best true discovery curve, followed by CircExplorer, while Find_circ2 and UROBORUS delivers the worst performance. CircScan discovers the largest number of true positives (*N* = 678), while Clirc reports only *N* = 357 true positives. However, Clirc achieves the best precision (0.371), a bit higher than the precision of CircScan (0.327); see Table 2. Find_circ2 identifies 386 true positives, slightly greater than the number identified by Clirc. However, it also identifies a huge amount of circRNAs (*N* = 88,325), making it the method with the lowest precision (0.004). Similarly, UROBORUS identifies a large number of circRNAs (*N* = 10113) with a small precision = 0.011. The remaining methods report only a few true positives (<30) across 108 samples; thus, they are not practically efficient for the CLIP-Seq data.

**Table 2:** **Results of all circRNA detection methods for the 108 CLIP-seq samples**

Method	# samples detected	# circRNAs detected	True positives	Precision
CircScan	53	2076	678	0.327
Clirc	69	962	357	0.371
CIRI2	10	51	13	0.255
CircExplorer	19	290	22	0.076
DCC	15	687	23	0.033
CircRNA_finder	24	4140	27	0.007
Find_circ2	108	88325	386	0.004
UROBORUS	56	10113	115	0.011

Only Find_circ2 reports circRNAs from all 108 CLIP-Seq samples, followed by Clirc, UROBORUS and CircScan with 69, 56 and 53 samples, respectively; the other methods identify circRNAs in less than <20*%* of the CLIP-Seq samples; see Table 2. Overall, these results from the CLIP-Seq data are in line with the findings of the short-read datasets in the simulation study.

Table S7 presents the performances of all the methods in individual CLIP-Seq protocols. CircScan obtains a higher precision (0.473) than the results of Clirc (0.379) in the largest group, PAR-CLIP, with N = 46 samples. However, Clirc outperforms CircScan in other big groups including iCLIP (*N* = 39), HITS-CLIP (*N* = 9), and irCLIP (*N* = 7). RNA-Seq based methods do not perform well for irCLIP even though all samples of the protocol contain long-read RNA-Seq data (with 76bp read length).

The Venn chart in Figure S23(a) shows a little overlap of detected circRNAs between CircScan, Clirc, UROBORUS and Find_circ2. More than half of the true positives identified by Find_circ2 and UROBORUS can be discovered by either CircScan or Clirc, as provided in Figure S23(b). The sets of overlapping circRNAs between the methods have better precision than the sets of distinct circRNAs of individual methods. For example, 107 of 135 circRNAs discovered by both Clirc and CircScan are true positives. Thus, the true positive rate (0.793) of the overlapping set between the two methods is two times greater than the overall precision of each method.

## Discussion and conclusion

In this study, we evaluate the performances of five RNA-Seq based methods and two CLIP-Seq based methods in the detection of circRNAs from both simulated and experimental SE RNA-Seq data. In the simulation study, we take into account the impacts of read length and the ratio of FP BSJs caused by tandem RNAs, and sequencing depth on the performances of the methods. For their assessment using real data, four experimental RNA-Seq datasets with long reads (≥100bp) and 124 CLIP-Seq samples with mostly short reads (≤50bp) from 45 different studies are utilized. A comparison with the results of PE RNA-Seq data is also done for the simulated datasets and the experimental RNA-Seq datasets.

The simulation study shows that increasing either read length or sequencing depth can improve the sensitivities of the methods for the SE RNA-Seq data. Only Clirc’s sensitivity reduces when the read length is greater than 75bp. The sensitivities are not greatly affected by the changes in the ratio of FP BSJs, indicating that most methods detect the BSJs efficiently. However, the ratio of FP BSJs significantly affects the precision of all methods with the simulated SE data. The PE mapping information indeed improves the precision of most RNA-Seq based methods for the simulated PE datasets. However, in the cases of the very long-read data (read length >150bp), CircExplorer, DDC, and CircRNA_finder show the substantial reduction of sensitivities and F1 scores with the PE data in the comparison with the SE data. Therefore, these methods perform better with the SE read rather than the PE read for the very long-read data. In contrast, UROBORUS performs better for the very-long PE read data, but it does not utilize well the PE mapping information for the shorter PE read data.

The results in this study are also concordant with the findings from the previous benchmark studies [21, 22] which are performed for PE long-read data. For example, we also observe that there no single method outperforms the others on all datasets. However, CIRI2 and CIRCexplorer are usually among the top-performing tools for the long-read SE data. Furthermore, there are significantly different performances between circRNA detection tools, especially in the CLIP-Seq datasets. For the long-read simulated datasets, Find_circ2 generally achieves the highest sensitivity and lowest precision; however, the performances of this method with the real datasets are highly dependent on the complexity of the data.

The CLIP-Seq based methods outperforms the RNA-seq based methods for both the short-read simulated datasets and the CLIP-Seq dataset. We note that the developers of RNA-Seq based methods might not have applied the methods for SE short-read RNA-Seq like the CLIP-Seq data, so their uses in this study are purely for comparative purposes. Neither Clirc nor CircScan is dominant for all datasets. Clirc is better than CircScan for the simulated data with read lengths of 42bp and 50bp but not 25bp. Regarding the CLIP-Seq protocols, CircScan outperforms Clirc in the PAR-LIP protocol, while Clirc performs better than CircScan in iCLIP and HITS-CLIP protocols. Find_circ2 outperforms the other RNA-Seq based methods with the short-read datasets. However, it reports a great number of false positives with the CLIP-seq datasets. In addition, integrating the results of CircScan, Clir, and Find_circ2 can significantly improve precision.

One of the study’s limitations is that we evaluated the circRNA detection methods based only on human data. Thus, performances of these methods on the SE RNA-Seq data of other species, such as mouse and plants, were not taken into account. Such an investigation is out of the scope of the current study and can be considered in the future. The validation of circRNAs using the RNase R+ samples in this study is widely used in circRNA studies, but it is not a perfect method. The digestion of linear RNAs in the RNase R+ samples does not always perform well [45]. Therefore, an independent validation for the detected circRNAs such as RT-PCR would help. A disadvantage of the RT-PCR approach is that it cannot be applied to a great number of circRNAs.

## Supplementary Information

**Additional file 1** The supplementary document contains all supplementary figures and tables.

## Data Availability

The command lines to run the circRNA detection tools, the codes and the results of simulated data to reproduce the figures and tables of this study on the Github site (https://github.com/hungnm805/SEcircRNAEvaluation) All experimental RNA-Seq datasets in this study are publicly available for download. Specifically, the links to download the RNA-Seq data of four cell lines in this study are https://trace.ncbi.nlm.nih.gov/Traces/sra/?study=SRP049453 for Hela and HEK293 cell lines, https://trace.ncbi.nlm.nih.gov/Traces/sra/?study=SRP011042 for Hs68 cell line, and https://trace.ncbi.nlm.nih.gov/Traces/sra/?study=SRP034543HEK293T cell line. The accession numbers of the samples used in each dataset are specifically provided in Supplementary Table S1. Supplementary Table S2 contains the accession numbers of samples and study IDs of 45 different studies of the CLIP-Seq dataset which can be also downloaded via the NCBI Sequence Read Archive (https://trace.ncbi.nlm.nih.gov/Traces/sra/).

## Abbreviations

RNA-Seq: RNA sequencing
BSJ: back-splicing junction
FP: false positive
circRNA: circular RNA
FDR: false discovery rate

## References

[1] Chen I, Chen C-Y, Chuang T-J (2015). Biogenesis, identification, and function of exonic circular RNAs. Wiley Interdiscip Rev RNA.

[2] Enuka Y, Lauriola M, Feldman ME, Sas-Chen A, Ulitsky I, Yarden Y (2016). Circular RNAs are long-lived and display only minimal early alterations in response to a growth factor. Nucleic Acids Res.

[3] Hansen TB, Jensen TI, Clausen BH, Bramsen JB, Finsen B, Damgaard CK, Kjems J (2013). Natural RNA circles function as efficient microRNA sponges. Nature.

[4] Peng L, Chen G, Zhu Z, Shen Z, Du C, Zang R, Su Y, Xie H, Li H, Xu X, Xia Y, Tang W (2016). Circular RNA ZNF609 functions as a competitive endogenous RNA to regulate AKT3 expression by sponging miR-150-5p in Hirschsprung’s disease. Oncotarget.

[5] Lu M (2020). Circular RNA: functions, applications and prospects. ExRNA.

[6] Qu S, Yang X, Li X, Wang J, Gao Y, Shang R, Sun W, Dou K, Li H (2015). Circular RNA: A new star of noncoding RNAs. Cancer Lett.

[7] Ashwal-Fluss R, Meyer M, Pamudurti NR, Ivanov A, Bartok O, Hanan M, Evantal N, Memczak S, Rajewsky N, Kadener S (2014). circRNA biogenesis competes with pre-mRNA splicing. Mol Cell.

[8] Li Z, Huang C, Bao C, Chen L, Lin M, Wang X, Zhong G, Yu B, Hu W, Dai L, Zhu P, Chang Z, Wu Q, Zhao Y, Jia Y, Xu P, Liu H, Shan G (2015). Exon-intron circular RNAs regulate transcription in the nucleus. Nat Struct Mol Biol.

[9] Zhang Y, Zhang X-O, Chen T, Xiang J-F, Yin Q-F, Xing Y-H, Zhu S, Yang L, Chen L-L (2013). Circular intronic long noncoding RNAs. Mol Cell.

[10] Zhang Z, Yang T, Xiao J (2018). Circular RNAs: Promising biomarkers for human diseases. EBioMedicine.

[11] Kukurba KR, Montgomery SB (2015). RNA sequencing and analysis. Cold Spring Harb Protoc.

[12] Zhao W, He X, Hoadley KA, Parker JS, Hayes DN, Perou CM (2014). Comparison of RNA-Seq by poly (A) capture, ribosomal RNA depletion, and DNA microarray for expression profiling. BMC Genomics.

[13] Corley SM, MacKenzie KL, Beverdam A, Roddam LF, Wilkins MR (2017). Differentially expressed genes from RNA-Seq and functional enrichment results are affected by the choice of single-end versus paired-end reads and stranded versus non-stranded protocols. BMC Genomics.

[14] Freedman AH, Gaspar JM, Sackton TB (2020). Short paired-end reads trump long single-end reads for expression analysis. BMC Bioinformatics.

[15] Chhangawala S, Rudy G, Mason CE, Rosenfeld JA (2015). The impact of read length on quantification of differentially expressed genes and splice junction detection. Genome Biol.

[16] Memczak S, Jens M, Elefsinioti A, Torti F, Krueger J, Rybak A, Maier L, Mackowiak SD, Gregersen LH, Munschauer M, Loewer A, Ziebold U, Landthaler M, Kocks C, le Noble F, Rajewsky N (2013). Circular RNAs are a large class of animal RNAs with regulatory potency. Nature.

[17] Änkö M-L, Neugebauer KM (2012). RNA–protein interactions in vivo: global gets specific. Trends Biochem Sci.

[18] Zhang M, Wang T, Xiao G, Xie Y (2020). Large-scale profiling of RBP-circRNA interactions from public CLIP-Seq datasets. Genes.

[19] Szabo L, Salzman J (2016). Detecting circular RNAs: bioinformatic and experimental challenges. Nat Rev Genet.

[20] Gao Y, Zhang J, Zhao F (2018). Circular RNA identification based on multiple seed matching. Brief Bioinform.

[21] Zeng X, Lin W, Guo M, Zou Q (2017). A comprehensive overview and evaluation of circular RNA detection tools. PLoS Comput Biol.

[22] Hansen TB, Venø MT, Damgaard CK, Kjems J (2016). Comparison of circular RNA prediction tools. Nucleic Acids Res.

[23] Hansen TB (2018). Improved circRNA identification by combining prediction algorithms. Front Cell Dev Biol.

[24] Zhang X-O, Wang H-B, Zhang Y, Lu X, Chen L-L, Yang L (2014). Complementary sequence-mediated exon circularization. Cell.

[25] Cheng J, Metge F, Dieterich C (2016). Specific identification and quantification of circular RNAs from sequencing data. Bioinformatics.

[26] Westholm JO, Miura P, Olson S, Shenker S, Joseph B, Sanfilippo P, Celniker SE, Graveley BR, Lai EC (2014). Genome-wide analysis of drosophila circular RNAs reveals their structural and sequence properties and age-dependent neural accumulation. Cell Rep.

[27] Song X, Zhang N, Han P, Moon B-S, Lai RK, Wang K, Lu W (2016). Circular RNA profile in gliomas revealed by identification tool UROBORUS. Nucleic Acids Res.

[28] Li B, Zhang X-Q, Liu S-R, Liu S, Sun W-J, Lin Q, Luo Y-X, Zhou K-R, Zhang C-M, Tan Y-Y, Yang J-H, Qu L-H. Discovering the Interactions between Circular RNAs and RNA-binding Proteins from CLIP-seq Data using circScan. bioRxiv. 2017:115980. 10.1101/115980. Accessed 09 Apr 2021.

[29] Chen L, Wang C, Sun H, Wang J, Liang Y, Wang Y, Wong G (2021). The bioinformatics toolbox for circRNA discovery and analysis. Brief Bioinform.

[30] Li H. Aligning sequence reads, clone sequences and assembly contigs with BWA-MEM. ArXiv. 2013; 1303.

[31] Gao Y, Zhao F (2018). Computational strategies for exploring circular RNAs. Trends Genet.

[32] Trapnell C, Pachter L, Salzberg SL (2009). TopHat: discovering splice junctions with RNA-Seq. Bioinformatics (Oxford, England).

[33] Langmead B, Trapnell C, Pop M, Salzberg SL (2009). Ultrafast and memory-efficient alignment of short DNA sequences to the human genome. Genome Biol.

[34] Wu TD, Nacu S (2010). Fast and SNP-tolerant detection of complex variants and splicing in short reads. Bioinformatics.

[35] Langmead B, Salzberg SL (2012). Fast gapped-read alignment with Bowtie 2. Nat Methods.

[36] Hafner M, Landthaler M, Burger L, Khorshid M, Hausser J, Berninger P, Rothballer A, Ascano M, Jungkamp A-C, Munschauer M, Ulrich A, Wardle GS, Dewell S, Zavolan M, Tuschl T (2010). Transcriptome-wide identification of RNA-binding protein and microRNA target sites by PAR-CLIP. Cell.

[37] Nguyen DT, Trac QT, Nguyen T-H, Nguyen H-N, Ohad N, Pawitan Y, Vu TN (2021). Circall: fast and accurate methodology for discovery of circular RNAs from paired-end RNA-sequencing data. BMC Bioinformatics.

[38] Glažar P, Papavasileiou P, Rajewsky N (2014). circBase: a database for circular RNAs. RNA (New York, N.Y.).

[39] Patro R, Duggal G, Love MI, Irizarry RA, Kingsford C (2017). Salmon provides fast and bias-aware quantification of transcript expression. Nat Methods.

[40] Frazee AC, Jaffe AE, Langmead B, Leek JT (2015). Polyester: simulating RNA-seq datasets with differential transcript expression. Bioinformatics (Oxford, England).

[41] Gao Y, Wang J, Zheng Y, Zhang J, Chen S, Zhao F (2016). Comprehensive identification of internal structure and alternative splicing events in circular RNAs. Nat Commun.

[42] Gao Y, Wang J, Zhao F (2015). CIRI: an efficient and unbiased algorithm for de novo circular RNA identification. Genome Biol.

[43] Jeck WR, Sorrentino JA, Wang K, Slevin MK, Burd CE, Liu J, Marzluff WF, Sharpless NE (2013). Circular RNAs are abundant, conserved, and associated with ALU repeats. RNA.

[44] Fan X, Zhang X, Wu X, Guo H, Hu Y, Tang F, Huang Y (2015). Single-cell RNA-seq transcriptome analysis of linear and circular RNAs in mouse preimplantation embryos. Genome Biol.

[45] Xiao M-S, Wilusz JE (2019). An improved method for circular RNA purification using RNase R that efficiently removes linear RNAs containing G-quadruplexes or structured 3’ ends. Nucleic Acids Res.

