# Supplementary material for: Evaluation of methods to detect circular RNAs from single-end RNA-sequencing data
Source: BMC Genomics. 2022 Feb 8;23:106. doi: 10.1186/s12864-022-08329-7 (PMC8822704; doi:10.1186/s12864-022-08329-7)
Supplement: Supplementary file 1 — Additional file 1 The supplementary document contains all supplementary figures and tables. [file 12864_2022_8329_MOESM1_ESM.pdf]

Supplementary document for Evaluation of methods to detect circular RNAs from single-end RNA-sequencing data

Manh Hung Nguyen, Ha-Nam Nguyen and Trung Nghia Vu

Command line for execution of circRNA detection methods

1 BWA

BWA aligner is used by CIRI2 and find_circ2 but with different parameters in their default pipeline.

1.1 Indexing genome

```
bwa index <FASTA genome>
```

2 STAR

STAR alignment is a part of CIRCExplorer, DCC and circRNA_finder pipelines. We used the same commands in the three tools' execution.

2.1 Indexing genome

```
STAR --runThreadN <num of threads> \
    --runMode genomeGenerate \
    --genomeDir <STAR index directory> \
    --genomeFastaFiles \
    --sjdbGTFfile <GTF annotation file> \
    --genomeSAsparseD 2 \
    --genomeSAindexNbases 14
```

2.2 Alignment (Single-end)

```
STAR --chimSegmentMin 20 \
    --runThreadN <num of threads> \
    --genomeDir <STAR index directory> \
    --readFilesIn <FASTQ SE sequence file> \
    --readFilesCommand zcat \
    --chimScoreMin 1 \
    --alignIntronMax 1000000 \
    --outFilterMismatchNoverReadLmax 0.02 \
    --alignTranscriptsPerReadNmax 100000 \
    --twopassMode Basic \
```

```
    --outSAMtype BAM SortedByCoordinate \
    --chimOutType Junctions SeparateSAMold \
    --outFilterMultimapNmax 2 \
    --limitBAMsortRAM 22298667641 \
    --outFileNamePrefix <STAR output path>
```

2.3 Alignment (Paired-end)

```
STAR --chimSegmentMin 20 \
    --runThreadN <num of threads> \
    --genomeDir <STAR index directory> \
    --readFilesIn <FASTQ PE sequence file - read 1> \
    <FASTQ PE sequence file - read 2> \
    --readFilesCommand zcat  \
    --chimScoreMin 1 \
    --alignIntronMax 1000000 \
    --outFilterMismatchNoverReadLmax 0.02 \
    --alignTranscriptsPerReadNmax 100000 \
    --twopassMode Basic --outSAMtype BAM SortedByCoordinate \
    --chimOutType Junctions SeparateSAMold \
    --outFilterMultimapNmax 2 \
    --limitBAMsortRAM 22298667641 \
    --outFileNamePrefix <STAR output path>
```

3 CIRI2

3.1 Single-end mode

```
bwa mem -t <num of threads> \
    <FASTA genome>  \
    <FASTQ SE sequence file> \
| perl CIRI2.pl \
    -I - \
    -O <CIRI result> \
    -F <FASTA genome> \
    -A <GTF annotation file> \
    -0 \
    -T <num of threads> \
```

3.2 Paired-end mode

```
bwa mem -t <num of threads> \
    <FASTA genome> \
    <FASTQ PE sequence file - read 1> \
    <FASTQ PE sequence file - read 2> \
| perl CIRI2.pl -I - \
    -O <CIRI result file> \
    -F <FASTA genome> \
    -A <GTF annotation file> \
    -0 \
```

```
    -T <num of threads>
```

4 CIRCExplorer

```
star_parse.py <STAR output path>/Chimeric.out.junction \
    <STAR output path>/fusion_junction.txt

CIRCexplorer.py -j <STAR output path>/fusion_junction.txt \
    -g \
    -r <CE txt reference> \
    -o <CIRCExplorer output path>
```

5 DCC

```
samtools index -b <STAR output path>/Aligned.sortedByCoord.out.bam \
    <STAR output path>/Aligned.sortedByCoord.out.bam.bai

DCC -T <num of threads> \
    <STAR output path>/Chimeric.out.junction \
    -D \
    -R <DCC repeat.gtf> \
    -an <GTF annotation file> \
    -B <STAR output path>/Aligned.sortedByCoord.out.bam \
    -F \
    -M \
    -Nr 1 1 \
    -fg \
    -G \
    -A \
    -O <DCC output directory>
```

6 CircRNA_finder

```
perl postProcessStarAlignment.pl --starDir <STAR output path> \
    --minLen 1 \
    --outDir <CF output path>
```

7 Find_circ2

7.1 Single-end mode

```
bwa mem -t<num of threads> \
    -A2 \
    -B10 \
    -k 15 \
    -T 1 \
    <FASTA genome> \
```

```
    <FASTQ SE sequence file> \
| find_circ.py --genome <FASTA genome> \
    -o <FC2 output directory>
```

7.2 Paired-end mode

```
bwa mem -t<num of threads> \
    -A2 \
    -B10 \
    -k 15 \
    -T 1 \
    <FASTA genome> \
    <FASTQ PE sequence file - read 1> \
    <FASTQ PE sequence file - read 2> \
| find_circ.py --genome <FASTA genome> \
    -o <FC2 output directory>
```

8 UROBORUS

8.1 Single-end mode

```
tophat --bowtie1  \
    -p <num of threads> \
    -o <UROBORUS output directory> \
    <bowtie1 genome index directory> \
    <FASTQ SE sequence file>

samtools view <UROBORUS output directory>/unmapped.bam \
    > <UROBORUS output directory>/unmapped.sam

perl UROBORUS.pl -p <num of threads> \
    -index <bowtie1 genome index directory> \
    -gtf <GTF annotation file> \
    -fasta <chromosomic genome directory> \
    <UROBORUS output directory>/unmapped.sam \
    <UROBORUS output directory>/accepted_hits.bam
```

8.2 Paired-end mode

```
tophat --bowtie1  \
    -p <num of threads> \
    -o <UROBORUS output directory> \
    <bowtie1 genome index directory> \
    <FASTQ PE sequence file - read 1> \
    <FASTQ PE sequence file - read 2> \

samtools view <UROBORUS output directory>/unmapped.bam \
    > <UROBORUS output directory>/unmapped.sam

perl UROBORUS.pl -p <num of threads> \
```

```
    -index <bowtie1 genome index directory> \
    -gtf <GTF annotation file> \
    -fasta <chromosomic genome directory> \
    <UROBORUS output directory>/unmapped.sam \
    <UROBORUS output directory>/accepted_hits.bam
```

9 Clirc

```
perl Clirc_library.pl -coord <CircBase List of circRNAs> \
    -genome <FASTA genome> \
    -library <Clirc library directory>

perl Clirc_search.pl -thread <num of threads> \
    -fastq <FASTQ SE sequence file> \
    -library <Clirc library directory> \
    -results <Clirc result file> \
    -cleanup

perl Clirc_filter.pl -out <Clirc result file> \
    -input <Clirc result file> \
    -library <Clirc library directory>
```

10 CircScan

```
bowtie2 -p <num of threads> \
    -t \
    -k 4 \
    --no-unal \
    -D 200 \
    -R 3 \
    -N 0 \
    -L 15 \
    -i S,1,0.5 \
    --score-min=C,-16,0 \
    -q \
    -U <FASTQ SE sequence file> \
    -x <bowtie2 genome index directory> \
    --un <bowtie2 alignment path>_minus.unmapped.fastq \
    > <bowtie2 alignment path>_minus.alignments.bwt2 \
    2> <bowtie2 alignment path>_minus.log

samtools view -bS <bowtie2 alignment path>_minus.alignments.bwt2 \
    |samtools sort - \
    |samtools index - > <bowtie2 alignment path>_minus.alignments.sorted.
       bam

bowtie2 -p <num of threads> \
    -t \
    --no-unal \
    -D 200 \
```

```
    -R 3 \
    -N 0 \
    -L 15 \
    -i S,1,0.5 \
    --score-min=C,-16,0 \
    -q \
    -U <bowtie2 alignment path>_minus.unmapped.fastq \
    -x <bowtie2 transcriptome index directory> \
    --un <bowtie2 alignment path>_minus.transcriptome.unmapped.fastq \
    <bowtie2 alignment path>_minus.transcriptome.alignments.bwt2

reformat.sh in=<bowtie2 alignment path>_minus.transcriptome.unmapped.fastq
    \
    out=<bowtie2 alignment path>_minus.transcriptome.unmapped.fa \
    overwrite=T

circScan <FASTA genome> \
    <Index file of FASTA genome> \
    <BED12 annotation> \
    <bowtie2 alignment path>_minus.alignments.sorted.bam \
    <bowtie2 alignment path>_minus.transcriptome.unmapped.fa \
    > <circScan result>
```

TABLES

Table S1: **Details of the experimental RNA-seq datasets.**

Cell-line	Dataset	Library size	Read length	SRA accession number
HEK293	RNase R-	31,059,167	150bp	SRR3479243
	RNase R+	42,307,449	150bp	SRR3449244
HELA	RNase R-	80,618,760	101bp	SRR1637089, SRR1637090
	RNase R+	36,815,458	101bp	SRR1636985, SRR1636986
HS68	RNase R-	206,362,733	100bp	SRR444975
	RNase R+	199,922,486	100bp	SRR445016
HEK293T	RNase R-	54,959,789	100bp	SRR1562287, SRR1567913, SRR1567914, SRR1567915
	RNase R+	35,281,556	100bp	SRR2048277, SRR2048278

Table S2: **List of 124 CLIP-seq samples from three cell lines HELA, HEK293 and HEK293T.**

Protocol	RBP	Study ID	Accession Number	Cell line	Library size	Read length	QC status
PAR-iCLIP	SRRM4	SRP041656	SRR1269756	HEK293T	45430635	50	Pass
PAR-iCLIP	PTBP1	SRP041656	SRR1269759	HEK293T	54721782	50	Pass
PAR-iCLIP	EIF3A	SRP064176	SRR2517447	HEK293T	27268730	51	Pass
PAR-iCLIP	EIF3A	SRP064176	SRR2517448	HEK293T	67751315	51	Pass
PAR-CLIP-MeRIP	HNRNPC	SRP040278	SRR1198832	HEK293T	48146267	50	Pass
PAR-CLIP-MeRIP	HNRNPC	SRP040278	SRR1198833	HEK293T	84729936	51	Pass
PAR-CLIP	IGF2BP1	SRP002487	SRR048951	HEK293	2313550	32	Pass
PAR-CLIP	IGF2BP1	SRP002487	SRR048952	HEK293	5088192	32	Pass
PAR-CLIP	IGF2BP1	SRP002487	SRR048956	HEK293	4454783	32	Pass
PAR-CLIP	IGF2BP2	SRP002487	SRR048957	HEK293	5424701	32	Pass
PAR-CLIP	IGF2BP2	SRP002487	SRR048958	HEK293	5096181	32	Pass
PAR-CLIP	IGF2BP2	SRP002487	SRR048959	HEK293	5048013	32	Pass
PAR-CLIP	IGF2BP3	SRP002487	SRR048962	HEK293	5528859	32	Pass
PAR-CLIP	IGF2BP3	SRP002487	SRR048963	HEK293	5139795	32	Pass
PAR-CLIP	IGF2BP3	SRP002487	SRR048964	HEK293	5305048	32	Pass
PAR-CLIP	AGO2	SRP002487	SRR048980	HEK293	4591805	32	Pass
PAR-CLIP	HNRNPC	SRP040278	SRR1198834	HEK293T	38249641	50	Pass
PAR-CLIP	HNRNPC	SRP040278	SRR1198835	HEK293T	41270877	50	Pass
PAR-CLIP	HNRNPC	SRP040278	SRR1198836	HEK293T	31199062	50	Pass
PAR-CLIP	HNRNPC	SRP040278	SRR1198837	HEK293T	50379364	50	Pass
PAR-CLIP	HNRNPC	SRP040278	SRR1198838	HEK293T	32914438	50	Pass
PAR-CLIP	HNRNPC	SRP040278	SRR1198839	HEK293T	35039022	50	Pass
PAR-CLIP	AGO2	SRP041231	SRR1241613	HEK293T	44512074	51	Pass
PAR-CLIP	YTHDC1	SRP043072	SRR1373360	HELA	25056247	37	Pass
PAR-CLIP	YTHDC1	SRP043072	SRR1373361	HELA	29776114	42	Pass
PAR-CLIP	WDR33	SRP046245	SRR1566437	HEK293	42006096	51	Pass
PAR-CLIP	YTHDF1	SRP050213	SRR1661485	HELA	35860487	30	Pass
PAR-CLIP	YTHDF1	SRP050213	SRR1661486	HELA	37561579	40	Pass
PAR-CLIP	ELAVL1	SRP006474	SRR189779	HEK293	50026686	36	Fail
PAR-CLIP	ELAVL1	SRP006474	SRR189780	HEK293	40672829	36	Pass
PAR-CLIP	ELAVL1	SRP006474	SRR189781	HEK293	55591375	36	Fail

Table S2 continued from previous page

Protocol	RBP	Study ID	Accession Number	Cell line	Library size	Read length	QC status
PAR-CLIP	AGO2	SRP006474	SRR189784	HEK293	31605425	38	Fail
PAR-CLIP	AGO2	SRP006474	SRR189786	HEK293	45468694	36	Pass
PAR-CLIP	YTHDC1	SRP061321	SRR2107149	HELA	84903973	101	Pass
PAR-CLIP	SRSF3	SRP061321	SRR2107151	HELA	80827271	101	Pass
PAR-CLIP	SRSF7	SRP061321	SRR2107152	HELA	93934006	101	Pass
PAR-CLIP	SRSF9	SRP061321	SRR2107153	HELA	77287159	101	Pass
PAR-CLIP	NUDT21	SRP012310	SRR488725	HEK293	20346443	38	Pass
PAR-CLIP	NUDT21	SRP012310	SRR488726	HEK293	11886294	38	Pass
PAR-CLIP	CPSF7	SRP012310	SRR488727	HEK293	57133855	36	Fail
PAR-CLIP	CPSF6	SRP012310	SRR488728	HEK293	24968982	38	Fail
PAR-CLIP	CPSF6	SRP012310	SRR488729	HEK293	33238836	38	Fail
PAR-CLIP	CPSF1	SRP012310	SRR488736	HEK293	35211644	38	Pass
PAR-CLIP	FIP1L1	SRP012310	SRR488739	HEK293	35402759	38	Fail
PAR-CLIP	CSTF2	SRP012310	SRR488740	HEK293	21240858	38	Pass
PAR-CLIP	CSTF2T	SRP012310	SRR488741	HEK293	53263321	36	Fail
PAR-CLIP	MOV10	SRP012463	SRR490650	HEK293	59512436	36	Fail
PAR-CLIP	CAPRIN1	SRP013363	SRR500485	HEK293	25130460	51	Pass
PAR-CLIP	ZC3H7B	SRP013363	SRR500487	HEK293	20860453	51	Pass
PAR-CLIP	AGO1	SRP018015	SRR650318	HEK293	28451979	36	Pass
PAR-CLIP	NOP58	SRP018104	SRR651722	HEK293	19552216	38	Fail
PAR-CLIP	NOP58	SRP018104	SRR651723	HEK293	18123257	38	Fail
PAR-CLIP	ELAVL1	SRP018242	SRR653239	HEK293T	155306420	46	Pass
PAR-CLIP	RBM10	SRP019250	SRR771562	HEK293	28803530	51	Pass
PAR-CLIP	RBM10	SRP019250	SRR771563	HEK293	32161258	51	Pass
PAR-CLIP	METTL3	SRP022152	SRR847347	HELA	23404657	100	Pass
PAR-CLIP	LIN28B	SRP022591	SRR850552	HEK293	7531061	51	Pass
PAR-CLIP	MOV10	SRP026278	SRR921441	HEK293	28929686	50	Pass
PAR-CLIP	MOV10	SRP026278	SRR921442	HEK293	21930363	50	Pass
PAR-CLIP	YTHDF2	SRP028325	SRR944646	HELA	35031546	28	Pass
PAR-CLIP	YTHDF2	SRP028325	SRR944647	HELA	25506982	100	Pass
PAR-CLIP	YTHDF2	SRP028325	SRR944648	HELA	32589998	100	Pass
PAR-CLIP	ELAVL1	SRP030015	SRR989637	HEK293	247835623	50	Pass
miCLIP	m6A	SRP050393	SRR2003406	HEK293	572850457	42	Pass
irCLIP	HNRNPC	SRP071075	SRR3199717	HELA	23543195	76	Pass
irCLIP	HNRNPC	SRP071075	SRR3199718	HELA	23116587	76	Pass
irCLIP	HNRNPC	SRP071075	SRR3199719	HELA	102591254	76	Pass
irCLIP	HNRNPC	SRP071075	SRR3199720	HELA	113301400	76	Pass
irCLIP	ELAVL1	SRP071075	SRR3199721	HELA	73076889	76	Pass
irCLIP	ELAVL1	SRP071075	SRR3199722	HELA	101258016	76	Pass
irCLIP	PTBP1	SRP071075	SRR3199724	HELA	68386825	76	Pass
iCLIP	RNPS1	ERP013821	ERR1201429	HELA	33715899	42	Pass
iCLIP	RNPS1	ERP013821	ERR1201430	HELA	29478768	42	Pass
iCLIP	RNPS1	ERP013821	ERR1201431	HELA	26188344	42	Pass
iCLIP	RNPS1	ERP013821	ERR1201432	HELA	23362640	34	Pass
iCLIP	RNPS1	ERP013821	ERR1201433	HELA	22691882	36	Pass
iCLIP	RNPS1	ERP013821	ERR1201434	HELA	18688782	41	Pass
iCLIP	RNPS1	ERP013821	ERR1201439	HELA	37127273	38	Pass
iCLIP	RNPS1	ERP013821	ERR1201440	HELA	28639999	35	Pass
iCLIP	RNPS1	ERP013821	ERR1201442	HELA	28329506	37	Pass
iCLIP	RNPS1	ERP013821	ERR1201444	HELA	34628259	42	Pass

Table S2 continued from previous page

Protocol	RBP	Study ID	Accession Number	Cell line	Library size	Read length	QC status
iCLIP	RNPS1	ERP013821	ERR1201445	HELA	34475502	37	Pass
iCLIP	RNPS1	ERP013821	ERR1201446	HELA	19156642	41	Pass
iCLIP	U2AF2	ERP001968	ERR196173	HELA	6215075	50	Pass
iCLIP	U2AF2	ERP001968	ERR196180	HELA	11312850	50	Pass
iCLIP	U2AF2	ERP001968	ERR196183	HELA	15512833	50	Pass
iCLIP	U2AF2	ERP001968	ERR196184	HELA	4372312	50	Pass
iCLIP	HNRNPC	ERP001968	ERR196189	HELA	7494419	50	Pass
iCLIP	U2AF2	ERP001968	ERR196190	HELA	2474111	50	Pass
iCLIP	UNK	ERP004684	ERR419264	HELA	23485238	91	Pass
iCLIP	EIF4A3	ERP005903	ERR514997	HELA	29781052	42	Pass
iCLIP	PTBP1	ERP005903	ERR514998	HELA	23501063	42	Pass
iCLIP	PTBP1	ERP008850	ERR689067	HELA	2201938	50	Pass
iCLIP	CSTF2T	SRP030467	SRR1002953	HELA	157717169	42	Pass
iCLIP	PTBP1	SRP041656	SRR1556935	HEK293T	73276360	50	Pass
iCLIP	PTBP1	SRP041656	SRR1556936	HEK293T	74729712	50	Pass
iCLIP	DDX21	SRP041180	SRR1910480	HEK293	74476092	75	Pass
iCLIP	DDX21	SRP041180	SRR1910481	HEK293	77668709	75	Pass
iCLIP	Acin1	SRP075202	SRR3509992	HELA	12473634	40	Pass
iCLIP	Acin1	SRP075202	SRR3509993	HELA	7054149	40	Pass
iCLIP	Acin1	SRP075202	SRR3509994	HELA	2377232	40	Pass
iCLIP	Acin1	SRP075202	SRR3509998	HELA	6343824	28	Pass
iCLIP	Acin1	SRP075202	SRR3509999	HELA	4144048	33	Pass
iCLIP	Acin1	SRP075202	SRR3510002	HELA	5423270	34	Pass
iCLIP	HNRNPL	SRP012488	SRR490912	HELA	13327397	50	Pass
iCLIP	HNRNPL	SRP012488	SRR490913	HELA	1220636	50	Pass
iCLIP	HNRNPL	SRP012488	SRR490914	HELA	1210822	50	Pass
iCLIP	CSTF2	SRP015741	SRR568009	HELA	23776796	42	Pass
iCLIP	CSTF2	SRP015741	SRR568010	HELA	29769986	42	Pass
iCLIP	CSTF2	SRP015741	SRR568011	HELA	31713229	42	Pass
iCLIP	UPF1	SRP026052	SRR901495	HELA	15418032	50	Fail
HITS-CLIP	DDX3X	SRP044063	SRR1507053	HEK293T	6366983	25	Pass
HITS-CLIP	METTL3	SRP045348	SRR1539123	HELA	27257968	43	Fail
HITS-CLIP	U2AF2	SRP047366	SRR1582592	HELA	31880691	36	Fail
HITS-CLIP	SLBP	SRP048566	SRR1594092	HELA	22414650	36	Pass
HITS-CLIP	SLBP	SRP048566	SRR1594093	HELA	23394224	36	Pass
HITS-CLIP	POLR2A	SRP051480	SRR1732275	HELA	5088901	49	Pass
HITS-CLIP	AGO2	SRP006474	SRR189782	HEK293	33271612	38	Fail
HITS-CLIP	UPF1	SRP059079	SRR2051421	HEK293T	130352798	50	Pass
HITS-CLIP	FBL	SRP068672	SRR3110177	HEK293	23514961	51	Pass
HITS-CLIP	HNRNPU	SRP009861	SRR390467	HELA	139623380	36	Fail
HITS-CLIP	DGCR8	SRP014009	SRR518498	HEK293T	36158041	49	Pass
HITS-CLIP	EIF4A3	SRP015711	SRR567525	HELA	32530810	80	Pass
HITS-CLIP	EIF4A3	SRP015711	SRR567526	HELA	33670095	80	Pass

Table S3: **Performances of all circRNA detection methods with the simulated SE datasets with the ratio setting 5-95 across read lengths.** In this simulation setting, 5% BSJs and 95% BSJs are expected from tandem RNAs and circRNAs, respectively. SimSE_25, SimSE_42, SimSE_50, SimSE_75, SimSE_100, SimSE_150, SimSE_200, SimSE_250 are the simulated SE data with the read length of 25, 42, 50, 75, 100, 150, 200 and 250bp, respectively.

Dataset	Method	# circRNAs detected	TP	FP	Sensitivity	Precision	F1
**SimSE_25**	CircScan	816	790	26	0.074	0.968	0.138
	Clirc	500	321	179	0.030	0.642	0.058
	CIRI2	0	0	0	0.000	0.000	0.000
	CircExplorer	0	0	0	0.000	0.000	0.000
	DCC	0	0	0	0.000	0.000	0.000
	CircRNA_finder	0	0	0	0.000	0.000	0.000
	Find_circ2	0	0	0	0.000	0.000	0.000
	UROBORUS	0	0	0	0.000	0.000	0.000
**SimSE_42**	CircScan	4246	4037	209	0.380	0.951	0.543
	Clirc	5663	5310	353	0.500	0.938	0.653
	CIRI2	0	0	0	0.000	0.000	0.000
	CircExplorer	0	0	0	0.000	0.000	0.000
	DCC	0	0	0	0.000	0.000	0.000
	CircRNA_finder	0	0	0	0.000	0.000	0.000
	Find_circ2	3861	3523	338	0.332	0.912	0.487
	UROBORUS	928	865	63	0.082	0.932	0.15
**SimSE_50**	CircScan	4833	4595	238	0.433	0.951	0.595
	Clirc	6226	5832	394	0.550	0.937	0.693
	CIRI2	0	0	0	0.000	0.000	0.000
	CircExplorer	2464	2406	58	0.227	0.976	0.368
	DCC	1747	1702	45	0.160	0.974	0.275
	CircRNA_finder	2404	2339	65	0.220	0.973	0.359
	Find_circ2	5065	4662	403	0.439	0.920	0.595
	UROBORUS	1406	1360	46	0.128	0.967	0.226
**SimSE_75**	CircScan	5823	5525	298	0.521	0.949	0.672
	Clirc	5593	5407	186	0.510	0.967	0.667
	CIRI2	5976	5794	182	0.546	0.970	0.699
	CircExplorer	5769	5600	169	0.528	0.971	0.684
	DCC	4607	4482	125	0.422	0.973	0.589
	CircRNA_finder	5609	5428	181	0.512	0.968	0.669
	Find_circ2	6911	6378	533	0.601	0.923	0.728
	UROBORUS	2201	2109	92	0.199	0.958	0.33
**SimSE_100**	CircScan	5780	5473	307	0.516	0.947	0.668
	Clirc	2628	2569	59	0.242	0.978	0.388
	CIRI2	7223	6994	229	0.659	0.968	0.784
	CircExplorer	6919	6704	215	0.632	0.969	0.765
	DCC	5743	5562	181	0.524	0.968	0.680
	CircRNA_finder	6721	6495	226	0.612	0.966	0.749
	Find_circ2	7786	7189	597	0.678	0.923	0.782
	UROBORUS	2674	2391	283	0.225	0.894	0.36
**SimSE_150**	CircScan	5906	5611	295	0.529	0.950	0.679
	Clirc	130	129	1	0.012	0.992	0.024
	CIRI2	8100	7802	298	0.735	0.963	0.834
	CircExplorer	7972	7699	273	0.726	0.966	0.829

	DCC	6739	6518	221	0.614	0.967	0.751
	CircRNA_finder	7735	7451	284	0.702	0.963	0.812
	Find_circ2	8750	8054	696	0.759	0.920	0.832
	UROBORUS	3504	2300	1204	0.217	0.656	0.326
**SimSE_200**	CircScan	5909	5607	302	0.528	0.949	0.679
	Clirc	130	129	1	0.012	0.992	0.024
	CIRI2	8495	8161	334	0.769	0.961	0.854
	CircExplorer	8490	8210	280	0.774	0.967	0.860
	DCC	7163	6937	226	0.654	0.968	0.781
	CircRNA_finder	8219	7941	278	0.748	0.966	0.843
	Find_circ2	8819	8115	704	0.765	0.920	0.835
	UROBORUS	3684	1437	2247	0.135	0.390	0.201
**SimSE_250**	CircScan	5953	5642	311	0.532	0.948	0.681
	Clirc	131	130	1	0.012	0.992	0.024
	CIRI2	8680	8343	337	0.786	0.961	0.865
	CircExplorer	8683	8386	297	0.790	0.966	0.869
	DCC	7269	7044	225	0.664	0.969	0.788
	CircRNA_finder	8400	8115	285	0.765	0.966	0.854
	Find_circ2	8764	8080	684	0.761	0.922	0.834
	UROBORUS	4011	1013	2998	0.095	0.253	0.139

Table S4: **Performances of all circRNA detection methods with the simulated SE datasets with the ratio setting 30-70 across read lengths.** In this simulation setting, 30% BSJs and 70% BSJs are expected from tandem RNAs and circRNAs, respectively. SimSE_25, SimSE_42, SimSE_50, SimSE_75, SimSE_100, SimSE_150, SimSE_200, SimSE_250 are the simulated SE data with the read length of 25, 42, 50, 75, 100, 150, 200 and 250bp, respectively.

**Dataset**	**Method**	**# circRNAs detected**	**TP**	**FP**	**Sensitivity**	**Precision**	**F1**
**SimSE_25**	CircScan	643	599	44	0.077	0.932	0.142
	Clirc	430	238	192	0.030	0.553	0.058
	CIRI2	0	0	0	0.000	0.000	0.000
	CircExplorer	0	0	0	0.000	0.000	0.000
	DCC	0	0	0	0.000	0.000	0.000
	CircRNA_finder	0	0	0	0.000	0.000	0.000
	Find_circ2	0	0	0	0.000	0.000	0.000
	UROBORUS	0	0	0	0.000	0.000	0.000
**SimSE_42**	CircScan	3576	2995	581	0.383	0.838	0.526
	Clirc	4892	3940	952	0.504	0.805	0.620
	CIRI2	0	0	0	0.000	0.000	0.000
	CircExplorer	0	0	0	0.000	0.000	0.000
	DCC	0	0	0	0.000	0.000	0.000
	CircRNA_finder	0	0	0	0.000	0.000	0.000
	Find_circ2	3289	2598	691	0.332	0.790	0.468
	UROBORUS	782	662	120	0.085	0.847	0.154
**SimSE_50**	CircScan	4122	3425	697	0.438	0.831	0.574
	Clirc	5393	4315	1078	0.552	0.800	0.653
	CIRI2	0	0	0	0.000	0.000	0.000
	CircExplorer	2040	1773	267	0.227	0.869	0.360
	DCC	1424	1268	156	0.162	0.890	0.274
	CircRNA_finder	1990	1726	264	0.221	0.867	0.352

	Find_circ2	4375	3454	921	0.442	0.789	0.566
	UROBORUS	1193	1029	164	0.132	0.863	0.228
**SimSE_75**	CircScan	5046	4096	950	0.524	0.812	0.637
	Clirc	4819	3984	835	0.509	0.827	0.630
	CIRI2	5206	4277	929	0.547	0.822	0.657
	CircExplorer	5018	4146	872	0.530	0.826	0.646
	DCC	3912	3312	600	0.423	0.847	0.565
	CircRNA_finder	4876	4017	859	0.514	0.824	0.633
	Find_circ2	6103	4709	1394	0.602	0.772	0.676
	UROBORUS	1907	1596	311	0.204	0.837	0.328
**SimSE_100**	CircScan	5011	4051	960	0.518	0.808	0.631
	Clirc	2140	1899	241	0.243	0.887	0.381
	CIRI2	6406	5172	1234	0.661	0.807	0.727
	CircExplorer	6087	4965	1122	0.635	0.816	0.714
	DCC	4961	4126	835	0.527	0.832	0.646
	CircRNA_finder	5909	4807	1102	0.615	0.814	0.700
	Find_circ2	6960	5310	1650	0.679	0.763	0.718
	UROBORUS	2347	1808	539	0.231	0.770	0.356
**SimSE_150**	CircScan	5079	4145	934	0.530	0.816	0.643
	Clirc	94	93	1	0.012	0.989	0.023
	CIRI2	7304	5772	1532	0.738	0.790	0.763
	CircExplorer	7121	5696	1425	0.728	0.800	0.762
	DCC	5881	4799	1082	0.614	0.816	0.700
	CircRNA_finder	6893	5512	1381	0.705	0.800	0.749
	Find_circ2	7918	5947	1971	0.760	0.751	0.756
	UROBORUS	3075	1717	1358	0.220	0.558	0.315
**SimSE_200**	CircScan	5107	4153	954	0.531	0.813	0.642
	Clirc	92	91	1	0.012	0.989	0.023
	CIRI2	7726	6042	1684	0.772	0.782	0.777
	CircExplorer	7620	6077	1543	0.777	0.798	0.787
	DCC	6240	5130	1110	0.656	0.822	0.730
	CircRNA_finder	7314	5880	1434	0.752	0.804	0.777
	Find_circ2	8000	6002	1998	0.767	0.750	0.759
	UROBORUS	3188	1055	2133	0.135	0.331	0.192
**SimSE_250**	CircScan	5121	4188	933	0.535	0.818	0.647
	Clirc	93	92	1	0.012	0.989	0.023
	CIRI2	7913	6155	1758	0.787	0.778	0.782
	CircExplorer	7826	6191	1635	0.791	0.791	0.791
	DCC	6312	5196	1116	0.664	0.823	0.735
	CircRNA_finder	7455	5990	1465	0.766	0.803	0.784
	Find_circ2	7949	5978	1971	0.764	0.752	0.758
	UROBORUS	3406	729	2677	0.093	0.214	0.130

Table S5: **Performances of all circRNA detection methods with the simulated SE datasets with the ratio setting 50-50 across read lengths.** In this simulation setting, 50% BSJs and 50% BSJs are expected from tandem RNAs and circRNAs, respectively. SimSE_25, SimSE_42, SimSE_50, SimSE_75, SimSE_100, SimSE_150, SimSE_200, SimSE_250 are the simulated SE data with the read length of 25, 42, 50, 75, 100, 150, 200 and 250bp, respectively.

Dataset	Method	# circRNAs detected	TP	FP	Sensitivity	Precision	F1
**SimSE_25**	CircScan	504	431	73	0.077	0.855	0.141
	Clirc	377	173	204	0.031	0.459	0.058
	CIRI2	0	0	0	0.000	0.000	0.000
	CircExplorer	0	0	0	0.000	0.000	0.000
	DCC	0	0	0	0.000	0.000	0.000
	CircRNA_finder	0	0	0	0.000	0.000	0.000
	Find_circ2	0	0	0	0.000	0.000	0.000
	UROBORUS	0	0	0	0.000	0.000	0.000
**SimSE_42**	CircScan	3056	2148	908	0.384	0.703	0.497
	Clirc	4233	2811	1422	0.503	0.664	0.572
	CIRI2	0	0	0	0.000	0.000	0.000
	CircExplorer	0	0	0	0.000	0.000	0.000
	DCC	0	0	0	0.000	0.000	0.000
	CircRNA_finder	0	0	0	0.000	0.000	0.000
	Find_circ2	2813	1868	945	0.334	0.664	0.445
	UROBORUS	648	470	178	0.084	0.725	0.151
**SimSE_50**	CircScan	3565	2453	1112	0.439	0.688	0.536
	Clirc	4756	3090	1666	0.553	0.650	0.597
	CIRI2	0	0	0	0.000	0.000	0.000
	CircExplorer	1713	1283	430	0.229	0.749	0.351
	DCC	1165	915	250	0.164	0.785	0.271
	CircRNA_finder	1670	1248	422	0.223	0.747	0.344
	Find_circ2	3852	2483	1369	0.444	0.645	0.526
	UROBORUS	1018	757	261	0.135	0.744	0.230
**SimSE_75**	CircScan	4394	2924	1470	0.523	0.665	0.586
	Clirc	4258	2849	1409	0.510	0.669	0.579
	CIRI2	4615	3063	1552	0.548	0.664	0.600
	CircExplorer	4419	2960	1459	0.529	0.670	0.591
	DCC	3390	2369	1021	0.424	0.699	0.528
	CircRNA_finder	4295	2867	1428	0.513	0.668	0.580
	Find_circ2	5430	3366	2064	0.602	0.620	0.611
	UROBORUS	1683	1150	533	0.206	0.683	0.316
**SimSE_100**	CircScan	4379	2903	1476	0.519	0.663	0.582
	Clirc	1756	1355	401	0.242	0.772	0.369
	CIRI2	5757	3687	2070	0.659	0.640	0.650
	CircExplorer	5412	3525	1887	0.630	0.651	0.641
	DCC	4339	2937	1402	0.525	0.677	0.592
	CircRNA_finder	5261	3413	1848	0.610	0.649	0.629
	Find_circ2	6299	3791	2508	0.678	0.602	0.638
	UROBORUS	2040	1311	729	0.234	0.643	0.344
**SimSE_150**	CircScan	4455	2975	1480	0.532	0.668	0.592
	Clirc	68	68	0	0.012	1.000	0.024
	CIRI2	6624	4132	2492	0.739	0.624	0.677
	CircExplorer	6393	4063	2330	0.727	0.636	0.678

	DCC	5170	3427	1743	0.613	0.663	0.637
	CircRNA_finder	6180	3933	2247	0.703	0.636	0.668
	Find_circ2	7195	4242	2953	0.759	0.590	0.664
	UROBORUS	2689	1241	1448	0.222	0.462	0.300
**SimSE_200**	CircScan	4459	2970	1489	0.531	0.666	0.591
	Clirc	67	67	0	0.012	1.000	0.024
	CIRI2	7071	4323	2748	0.773	0.611	0.683
	CircExplorer	6878	4335	2543	0.775	0.630	0.695
	DCC	5516	3669	1847	0.656	0.665	0.661
	CircRNA_finder	6562	4195	2367	0.750	0.639	0.690
	Find_circ2	7265	4278	2987	0.765	0.589	0.666
	UROBORUS	2792	773	2019	0.138	0.277	0.184
**SimSE_250**	CircScan	4440	2987	1453	0.534	0.673	0.596
	Clirc	68	68	0	0.012	1.000	0.024
	CIRI2	7301	4403	2898	0.788	0.603	0.683
	CircExplorer	7128	4434	2694	0.793	0.622	0.697
	DCC	5566	3720	1846	0.665	0.668	0.667
	CircRNA_finder	6700	4287	2413	0.767	0.640	0.698
	Find_circ2	7220	4259	2961	0.762	0.590	0.665
	UROBORUS	2940	528	2412	0.094	0.180	0.124

Table S6: **Performances of all circRNA detection methods with the simulated SE datasets with the ratio setting 80-20 across read lengths.** In this simulation setting, 80% BSJs and 20% BSJs are expected from tandem RNAs and circRNAs, respectively. SimSE_25, SimSE_42, SimSE_50, SimSE_75, SimSE_100, SimSE_150, SimSE_200, SimSE_250 are the simulated SE data with the read length of 25, 42, 50, 75, 100, 150, 200 and 250bp, respectively.

Dataset	Method	# circRNAs detected	TP	FP	Sensitivity	Precision	F1
**SimSE_25**	CircScan	268	163	105	0.073	0.608	0.130
	Clirc	302	61	241	0.027	0.202	0.048
	CIRI2	0	0	0	0.000	0.000	0.000
	CircExplorer	0	0	0	0.000	0.000	0.000
	DCC	0	0	0	0.000	0.000	0.000
	CircRNA_finder	0	0	0	0.000	0.000	0.000
	Find_circ2	0	0	0	0.000	0.000	0.000
	UROBORUS	0	0	0	0.000	0.000	0.000
**SimSE_42**	CircScan	2247	865	1382	0.385	0.385	0.385
	Clirc	3290	1118	2172	0.498	0.340	0.404
	CIRI2	0	0	0	0.000	0.000	0.000
	CircExplorer	0	0	0	0.000	0.000	0.000
	DCC	0	0	0	0.000	0.000	0.000
	CircRNA_finder	0	0	0	0.000	0.000	0.000
	Find_circ2	2112	746	1366	0.332	0.353	0.343
	UROBORUS	480	200	280	0.089	0.417	0.147
**SimSE_50**	CircScan	2694	998	1696	0.445	0.370	0.404
	Clirc	3726	1234	2492	0.550	0.331	0.413
	CIRI2	0	0	0	0.000	0.000	0.000
	CircExplorer	1210	505	705	0.225	0.417	0.292
	DCC	783	364	419	0.162	0.465	0.241
	CircRNA_finder	1170	489	681	0.218	0.418	0.286
	Find_circ2	2983	984	1999	0.439	0.330	0.377
	UROBORUS	739	293	446	0.131	0.396	0.196
**SimSE_75**	CircScan	3448	1161	2287	0.517	0.337	0.408
	Clirc	3356	1136	2220	0.506	0.338	0.406
	CIRI2	3730	1234	2496	0.550	0.331	0.413
	CircExplorer	3509	1176	2333	0.524	0.335	0.409
	DCC	2587	942	1645	0.420	0.364	0.390
	CircRNA_finder	3406	1134	2272	0.505	0.333	0.401
	Find_circ2	4448	1335	3113	0.595	0.300	0.399
	UROBORUS	1297	442	855	0.197	0.341	0.250
**SimSE_100**	CircScan	3434	1170	2264	0.521	0.341	0.412
	Clirc	1172	522	650	0.233	0.445	0.306
	CIRI2	4831	1492	3339	0.665	0.309	0.422
	CircExplorer	4424	1418	3006	0.632	0.321	0.425
	DCC	3354	1186	2168	0.529	0.354	0.424
	CircRNA_finder	4295	1367	2928	0.609	0.318	0.418
	Find_circ2	5316	1527	3789	0.680	0.287	0.404
	UROBORUS	1634	502	1132	0.224	0.307	0.259
**SimSE_150**	CircScan	3502	1186	2316	0.529	0.339	0.413
	Clirc	28	28	0	0.012	1.000	0.025
	CIRI2	5733	1677	4056	0.747	0.293	0.420
	CircExplorer	5427	1648	3779	0.734	0.304	0.430

	DCC	4187	1385	2802	0.617	0.331	0.431
	CircRNA_finder	5219	1591	3628	0.709	0.305	0.426
	Find_circ2	6300	1714	4586	0.764	0.272	0.401
	UROBORUS	2100	460	1640	0.205	0.219	0.212
**SimSE_200**	CircScan	3471	1189	2282	0.530	0.343	0.416
	Clirc	28	28	0	0.012	1.000	0.025
	CIRI2	6100	1743	4357	0.777	0.286	0.418
	CircExplorer	5821	1743	4078	0.777	0.299	0.432
	DCC	4434	1469	2965	0.655	0.331	0.440
	CircRNA_finder	5448	1682	3766	0.750	0.309	0.437
	Find_circ2	6290	1735	4555	0.773	0.276	0.407
	UROBORUS	2093	289	1804	0.129	0.138	0.133
**SimSE_250**	CircScan	3450	1189	2261	0.530	0.345	0.418
	Clirc	28	28	0	0.012	1.000	0.025
	CIRI2	6432	1779	4653	0.793	0.277	0.410
	CircExplorer	6113	1792	4321	0.799	0.293	0.429
	DCC	4459	1494	2965	0.666	0.335	0.446
	CircRNA_finder	5595	1724	3871	0.768	0.308	0.440
	Find_circ2	6204	1720	4484	0.766	0.277	0.407
	UROBORUS	2177	192	1985	0.856	0.088	0.087

Table S7: **Results of all circRNA detection methods for the CLIP-seq dataset across protocols.**

Protocol	Method	# samples detected	# circRNAs detected	True positives	Precision
PAR-CLIP	CircScan	20	184	87	0.473
	Clirc	26	116	44	0.379
	CIRI2	3	15	0	0.000
	CircExplorer	3	5	2	0.400
	DCC	5	70	4	0.057
	CircRNA_finder	8	76	4	0.053
	Find_circ2	46	16855	41	0.002
	UROBORUS	11	162	14	0.086
iCLIP	CircScan	24	1521	449	0.295
	Clirc	29	765	282	0.369
	CIRI2	2	9	1	0.111
	CircExplorer	2	45	2	0.044
	DCC	1	61	2	0.033
	CircRNA_finder	2	165	4	0.024
	Find_circ2	39	15059	178	0.012
	UROBORUS	33	8958	86	0.010
HITS-CLIP	CircScan	7	315	110	0.349
	Clirc	6	53	27	0.509
	CIRI2	2	24	12	0.500
	CircExplorer	5	64	16	0.250
	DCC	5	84	16	0.190
	CircRNA_finder	6	89	16	0.180
	Find_circ2	9	2393	114	0.048
	UROBORUS	5	397	12	0.030
irCLIP	CircScan	0	0	0	NA
	Clirc	2	5	1	0.200
	CIRI2	3	3	0	0.000
	CircExplorer	7	172	1	0.006
	DCC	3	467	0	0.000
	CircRNA_finder	7	3803	1	0.000
	Find_circ2	7	44968	24	0.001
	UROBORUS	0	0	0	NA
PAR-iCLIP	CircScan	0	0	0	NA
	Clirc	4	11	0	0.000
	CIRI2	0	0	0	NA
	CircExplorer	0	0	0	NA
	DCC	0	0	0	NA
	CircRNA_finder	0	0	0	NA
	Find_circ2	4	3741	12	0.003
	UROBORUS	4	353	0	0.000
PAR-CLIP-MeRIP	CircScan	1	24	14	0.583
	Clirc	2	12	3	0.250
	CIRI2	0	0	0	NA
	CircExplorer	2	4	1	0.250
	DCC	1	5	1	0.200
	CircRNA_finder	1	7	2	0.286
	Find_circ2	2	5240	10	0.002
	UROBORUS	2	59	1	0.017

miCLIP	CircScan	1	32	18	0.562
	Clirc	0	0	0	NA
	CIRI2	0	0	0	NA
	CircExplorer	0	0	0	NA
	DCC	0	0	0	NA
	CircRNA_finder	0	0	0	NA
	Find_circ2	1	69	7	0.101
	UROBORUS	1	184	2	0.011

FIGURES

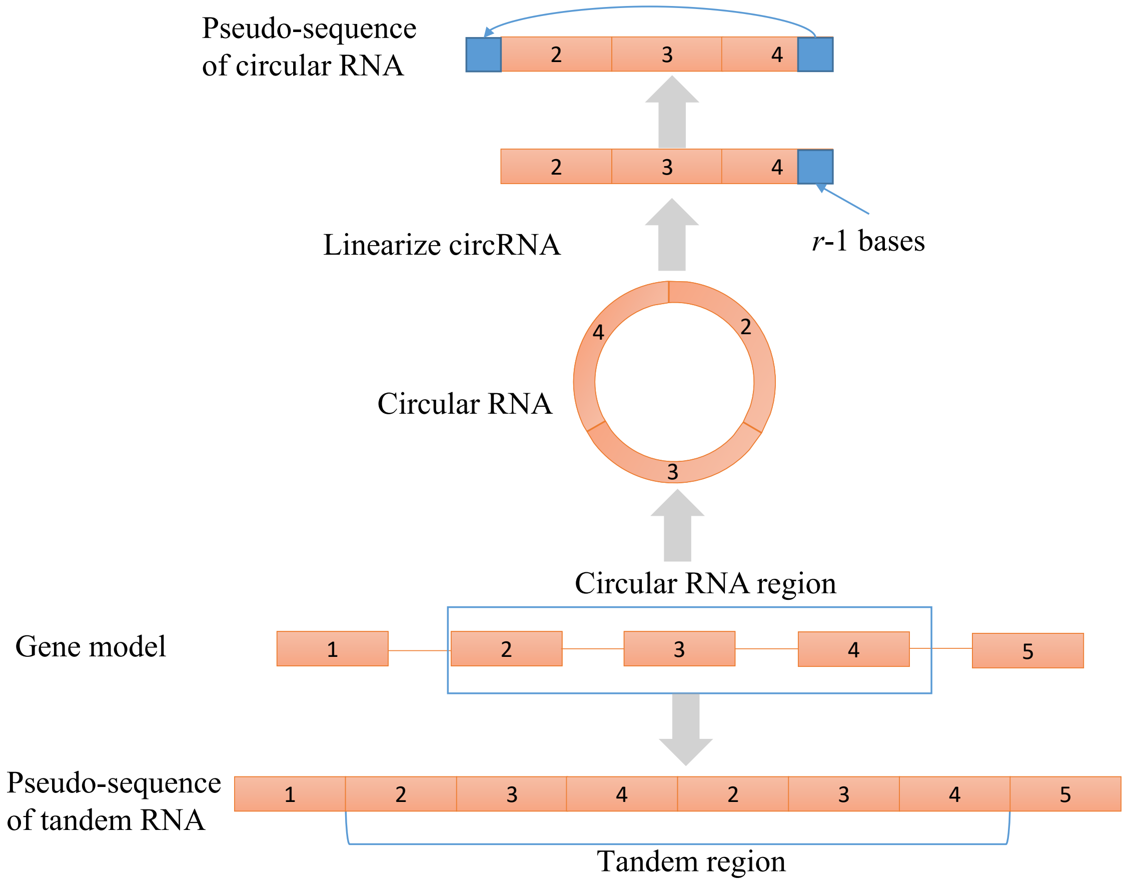

Figure S1: **Generation of the pseudo-sequences of circRNAs and tandem RNAs for simulation.** For circRNAs, the circular transcript is linearized then r-1 last bases are copied to the start of the linearized transcript to build the pseudo-sequence, where r is the read length. For tandem RNA, we duplicate the circRNA region for the pseudo sequence.

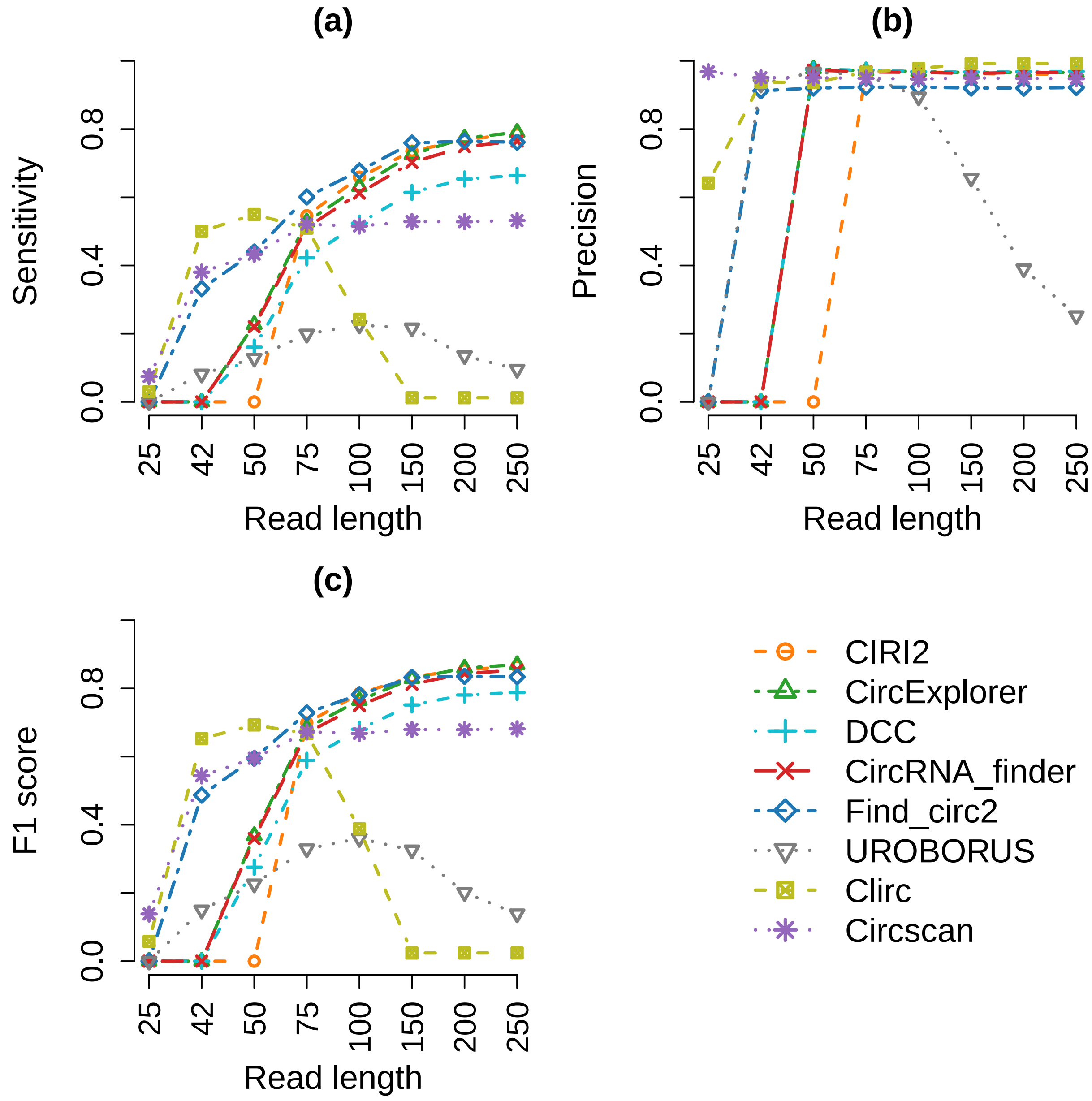

Figure S2: **Performances of all circRNA detection methods in the simulated SE datasets of the ratio setting 5-95 across read lengths.** The x-axis presents the read length of the simulated samples. The y-axis in panels (a), (b), and (c) presents sensitivity, precision, and F1 score, respectively.

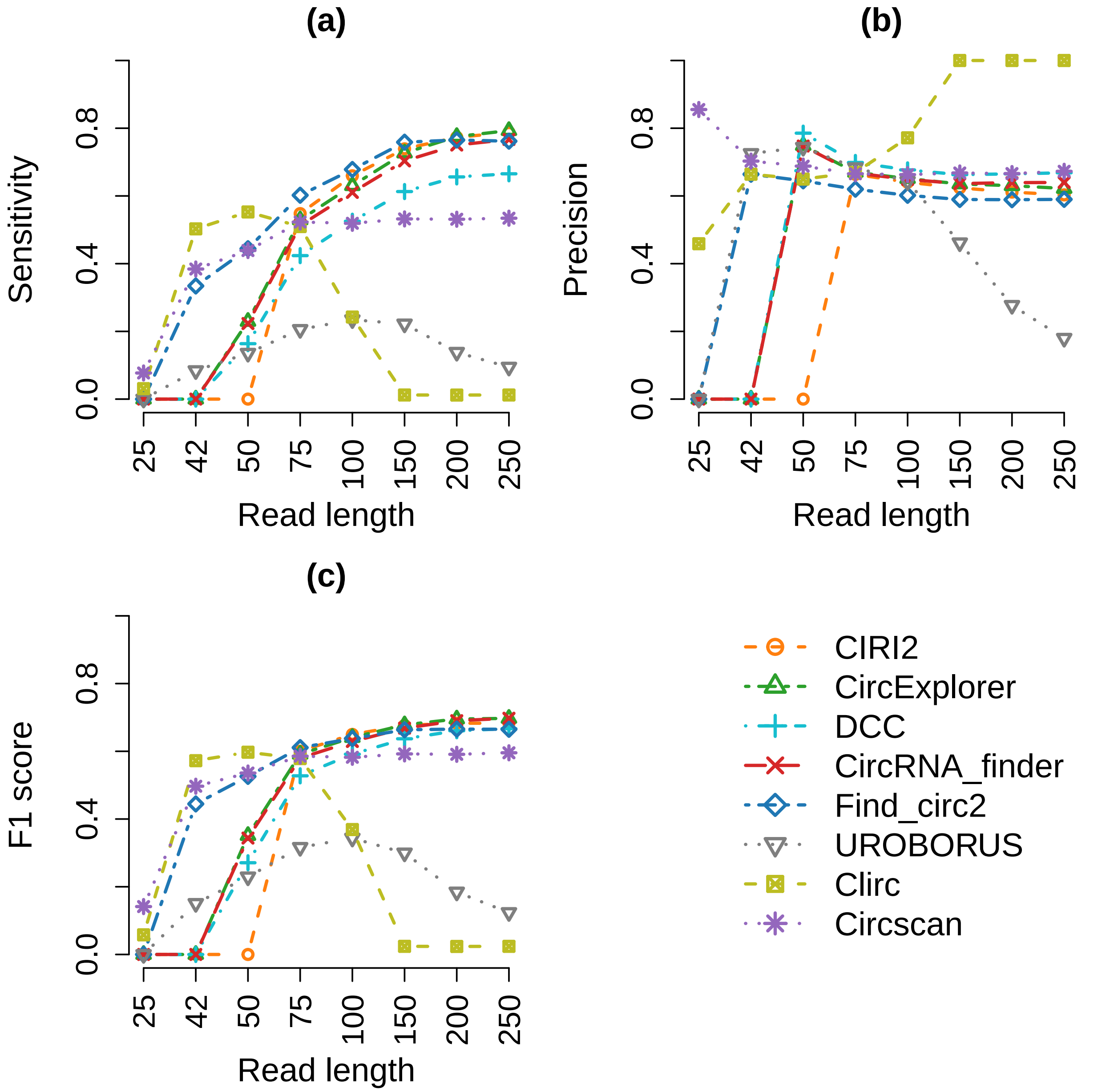

Figure S3: **Performances of all circRNA detection methods in the simulated SE datasets of the ratio setting 50-50 across read lengths.** The x-axis presents the read length of the simulated samples. The y-axis in panels (a), (b), and (c) presents sensitivity, precision, and F1 score, respectively.

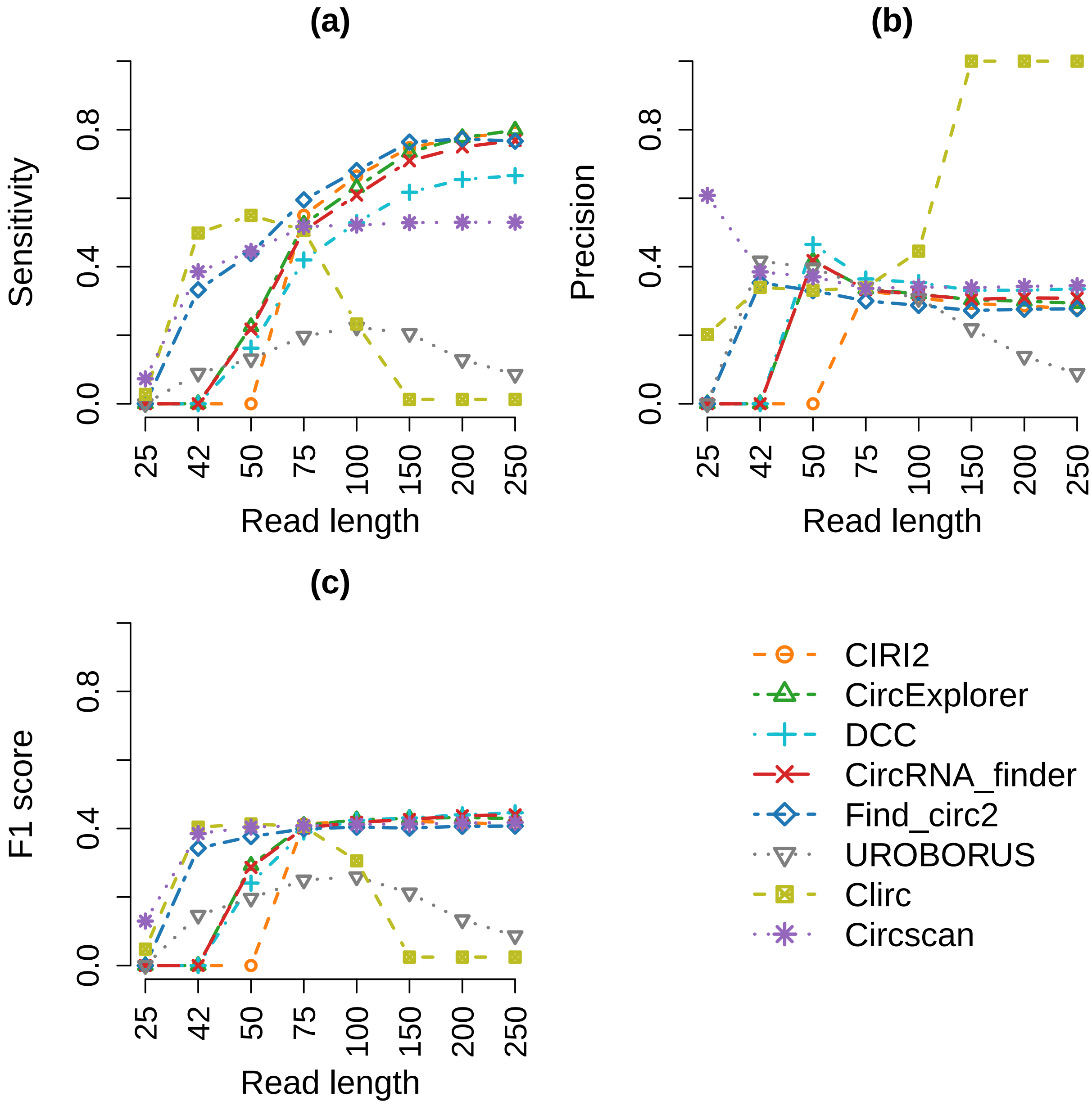

Figure S4: **Performances of all circRNA detection methods in the simulated SE datasets of the ratio setting 80-20 across read lengths.** The x-axis presents the read length of the simulated samples. The y-axis in panels (a), (b), and (c) presents sensitivity, precision, and F1 score, respectively.

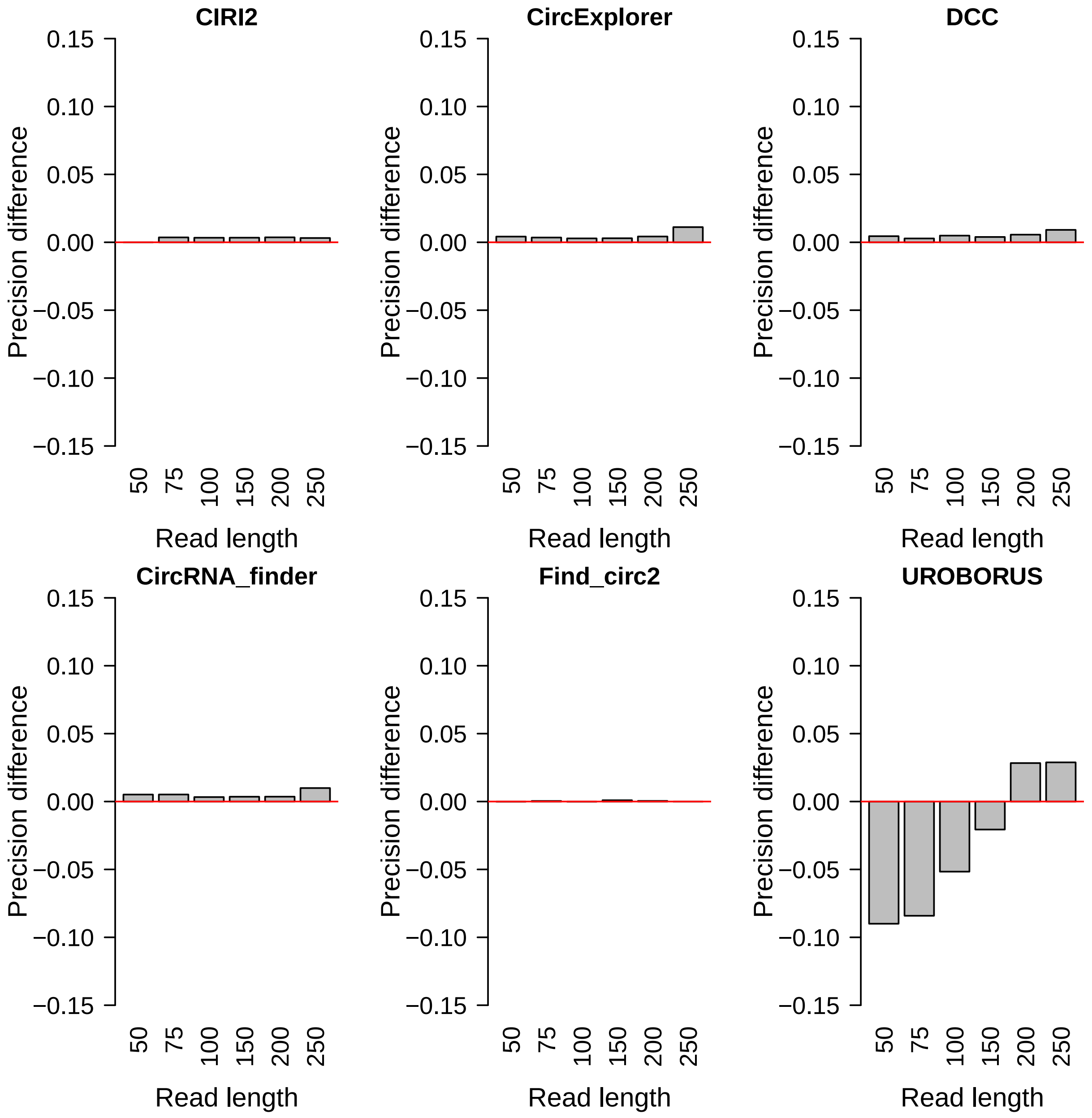

Figure S5: **The difference of precision between the results of the simulated PE and SE datasets in the ratio setting of 5-95.** The x-axis presents the read length of the simulated samples. The y-axis shows the precision difference. A positive (negative) difference indicates the precision from the PE dataset is greater (less) than that of the SE dataset.

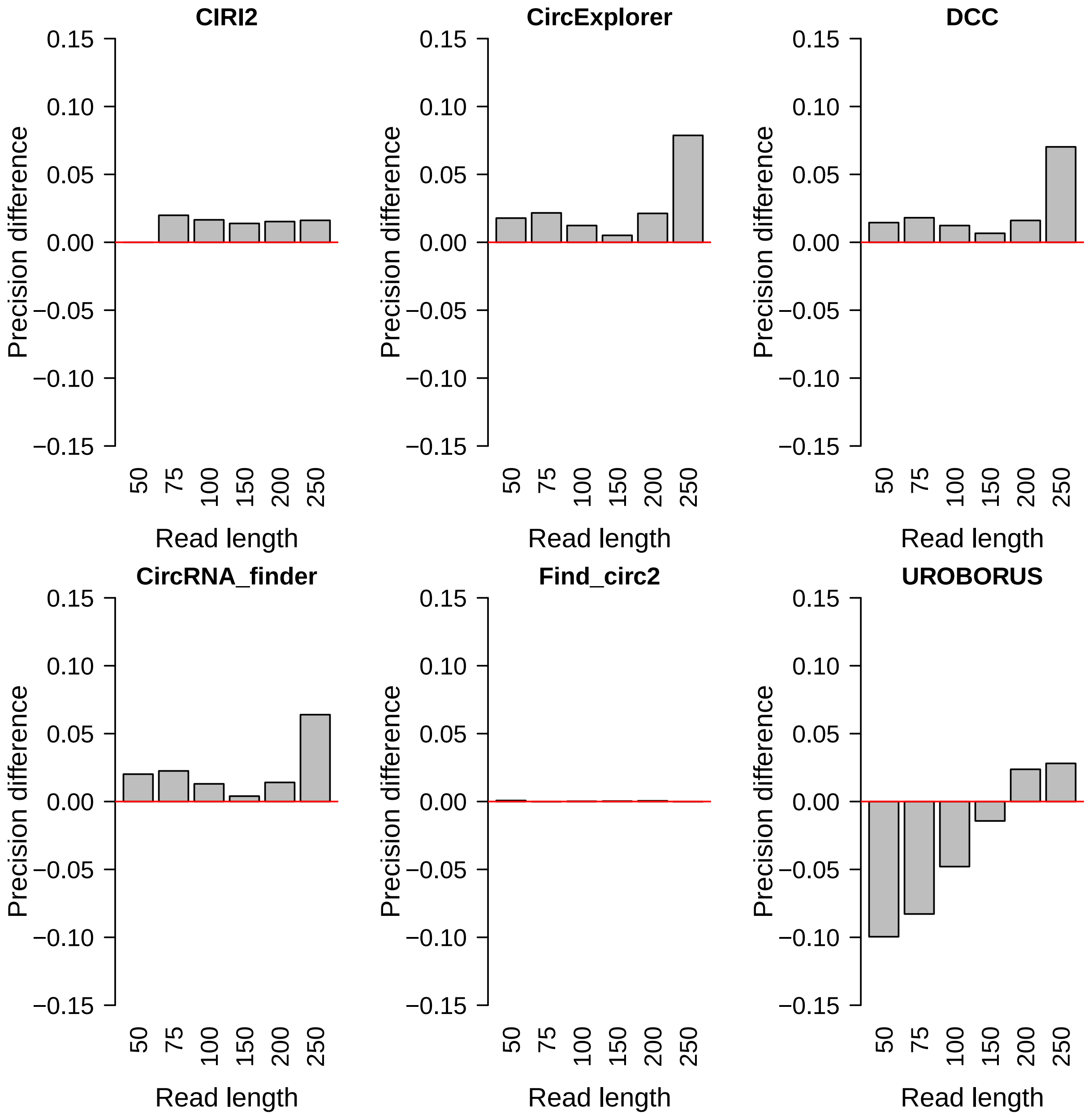

Figure S6: **The difference of precision between the results of the simulated PE and SE datasets in the ratio setting of 30-70.** The x-axis presents the read length of the simulated samples. The y-axis shows the precision difference. A positive (negative) difference indicates the precision from the PE dataset is greater (less) than that of the SE dataset.

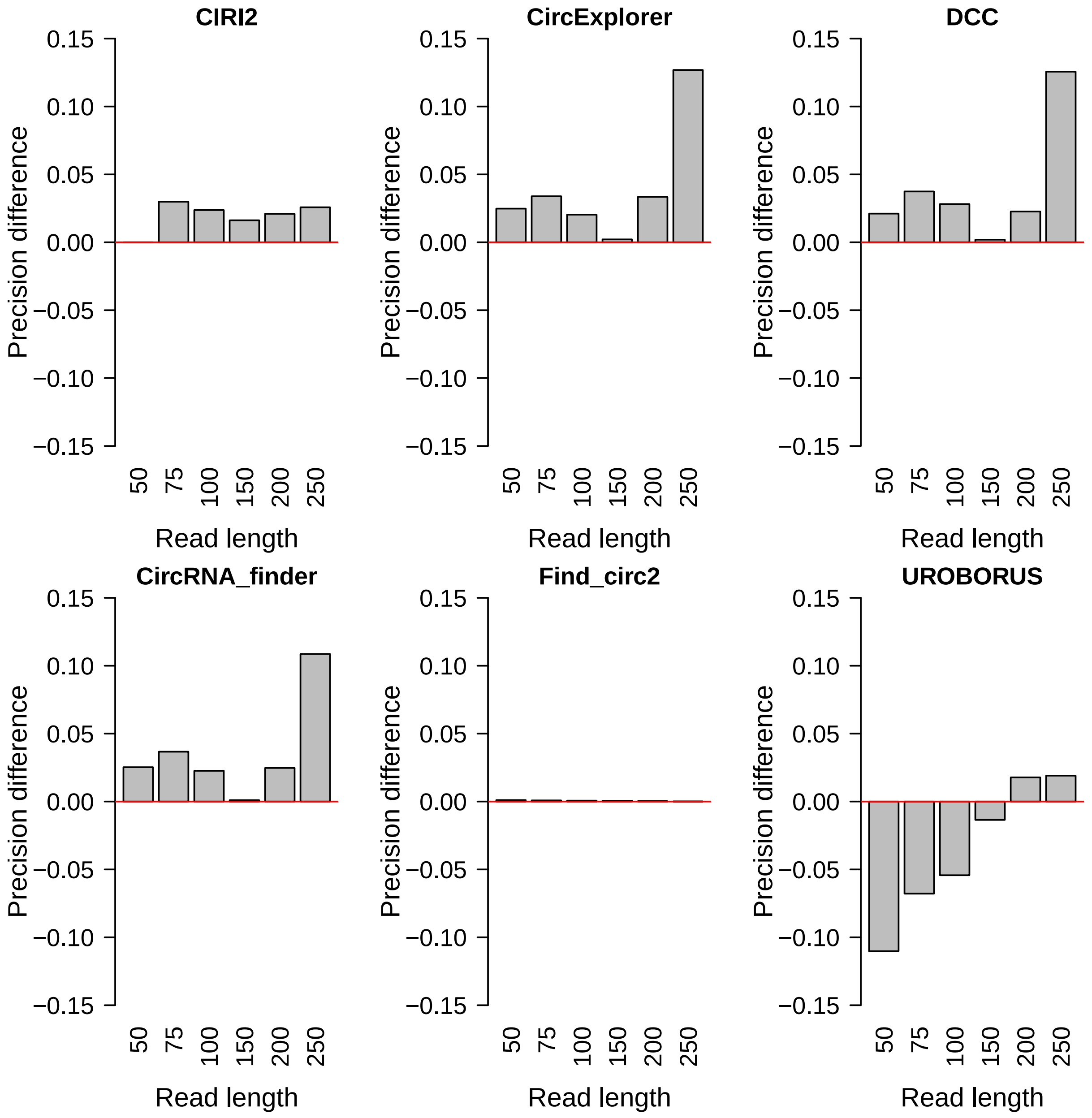

Figure S7: **The difference of precision between the results of the simulated PE and SE datasets in the ratio setting of 50-50.** The x-axis presents the read length of the simulated samples. The y-axis shows the precision difference. A positive (negative) difference indicates the precision from the PE dataset is greater (less) than that of the SE dataset.

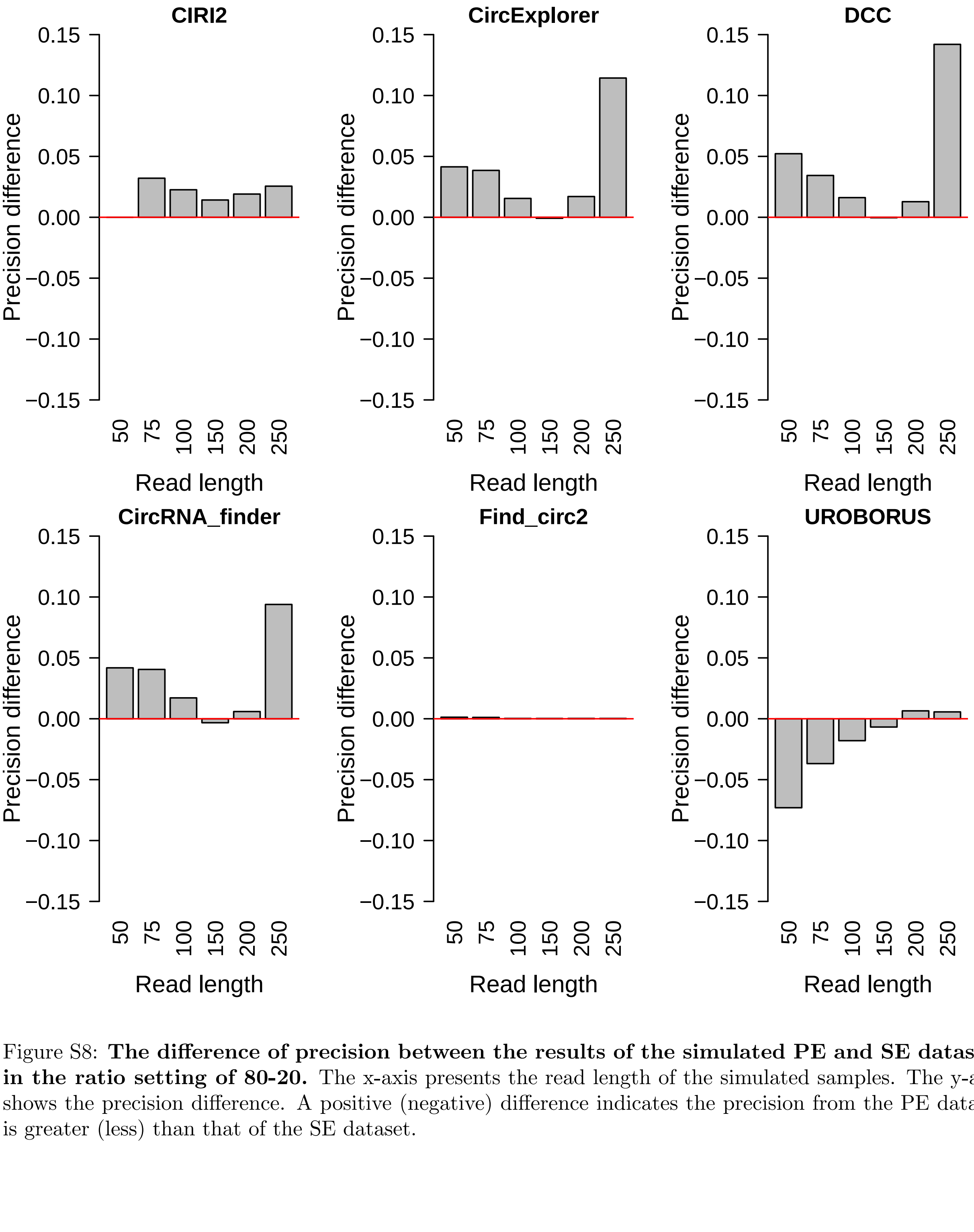

Figure S8: **The difference of precision between the results of the simulated PE and SE datasets in the ratio setting of 80-20.** The x-axis presents the read length of the simulated samples. The y-axis shows the precision difference. A positive (negative) difference indicates the precision from the PE dataset is greater (less) than that of the SE dataset.

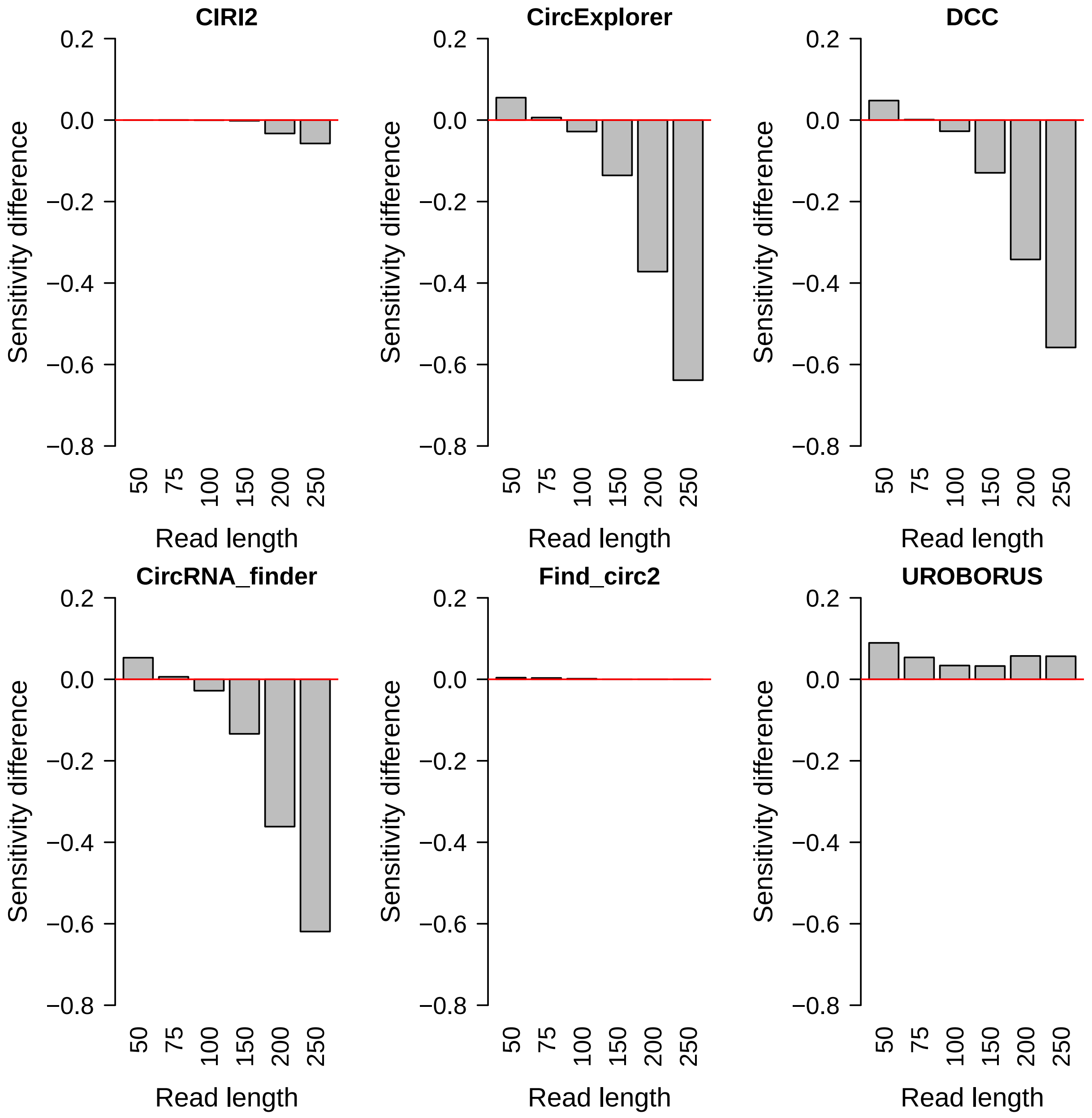

Figure S9: **The difference of sensitivity between the results of the PE and simulated SE datasets in the ratio setting of 5-95.** The x-axis presents the read length of the simulated samples. The y-axis shows the sensitivity difference. A positive (negative) difference indicates the sensitivity from the PE dataset is greater (less) than that of the SE dataset.

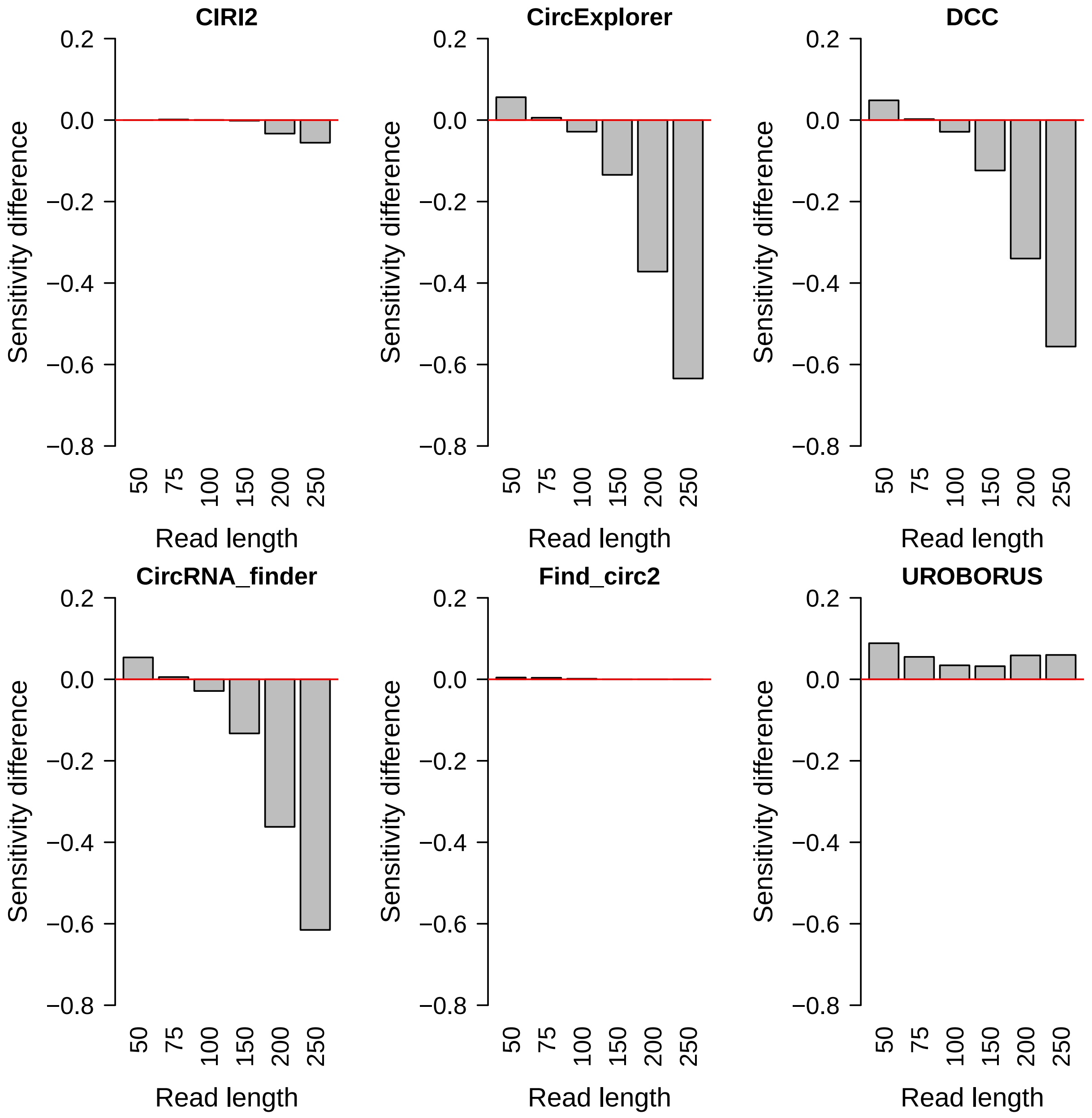

Figure S10: **The difference of sensitivity between the results of the simulated PE and SE datasets in the ratio setting of 30-70.** The x-axis presents the read length of the simulated samples. The y-axis shows the sensitivity difference. A positive (negative) difference indicates the sensitivity from the PE dataset is greater (less) than that of the SE dataset.

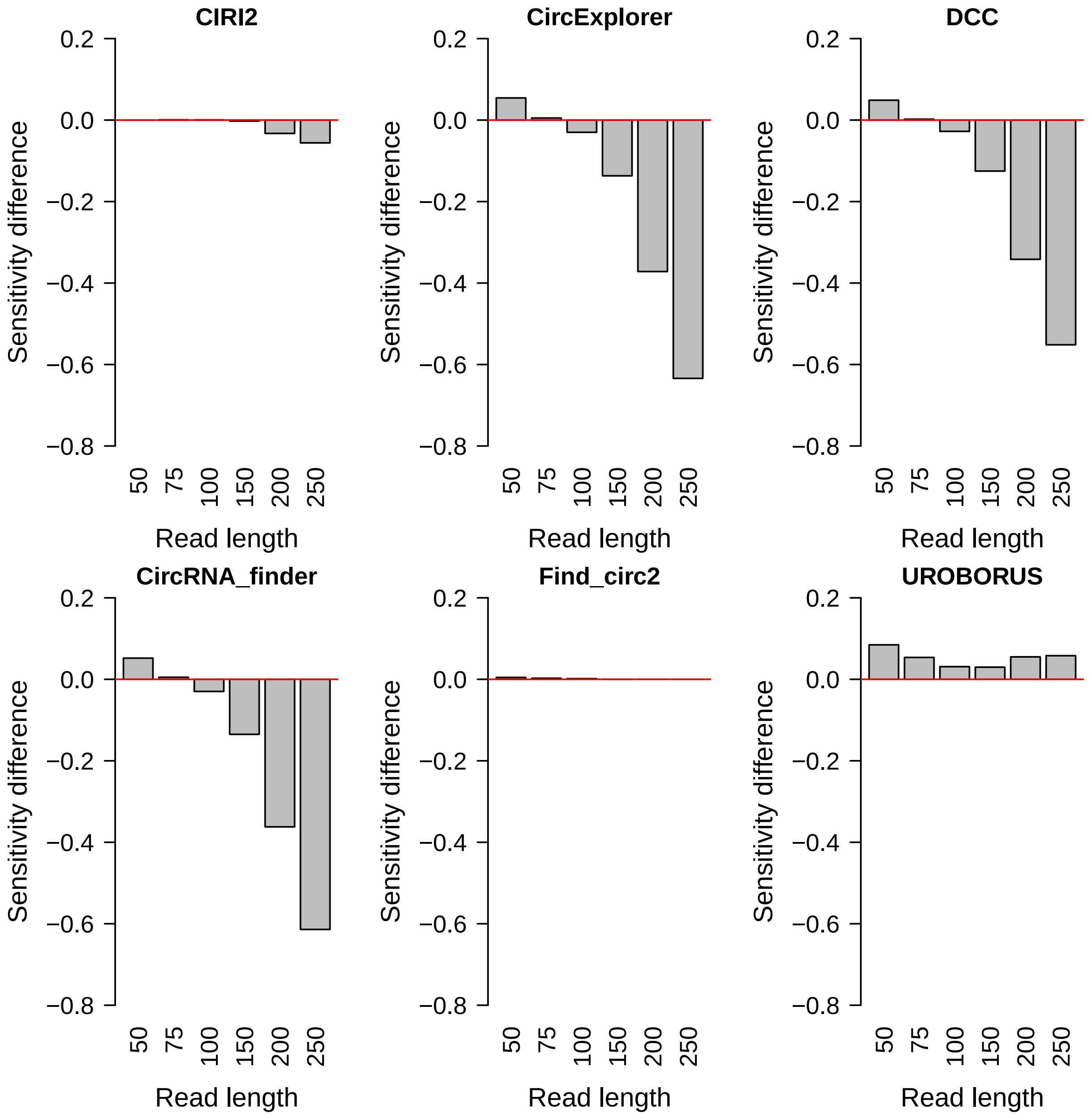

Figure S11: **The difference of sensitivity between the results of the simulated PE and SE datasets in the ratio setting of 50-50.** The x-axis presents the read length of the simulated samples. The y-axis shows the sensitivity difference. A positive (negative) difference indicates the sensitivity from the PE dataset is greater (less) than that of the SE dataset.

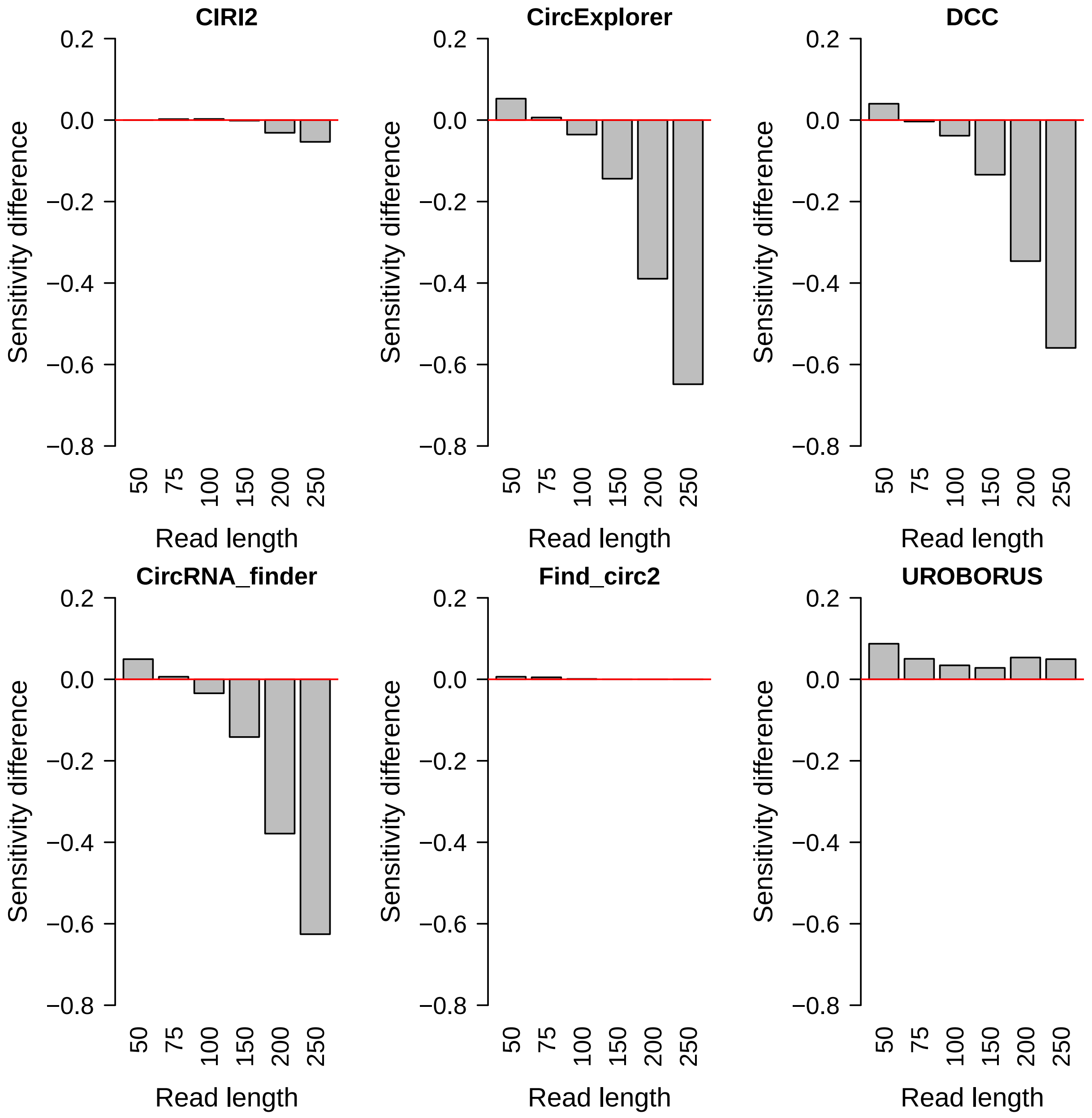

Figure S12: **The difference of sensitivity between the results of the simulated PE and SE datasets in the ratio setting of 80-20.** The x-axis presents the read length of the simulated samples. The y-axis shows the sensitivity difference. A positive (negative) difference indicates the sensitivity from the PE dataset is greater (less) than that of the SE dataset.

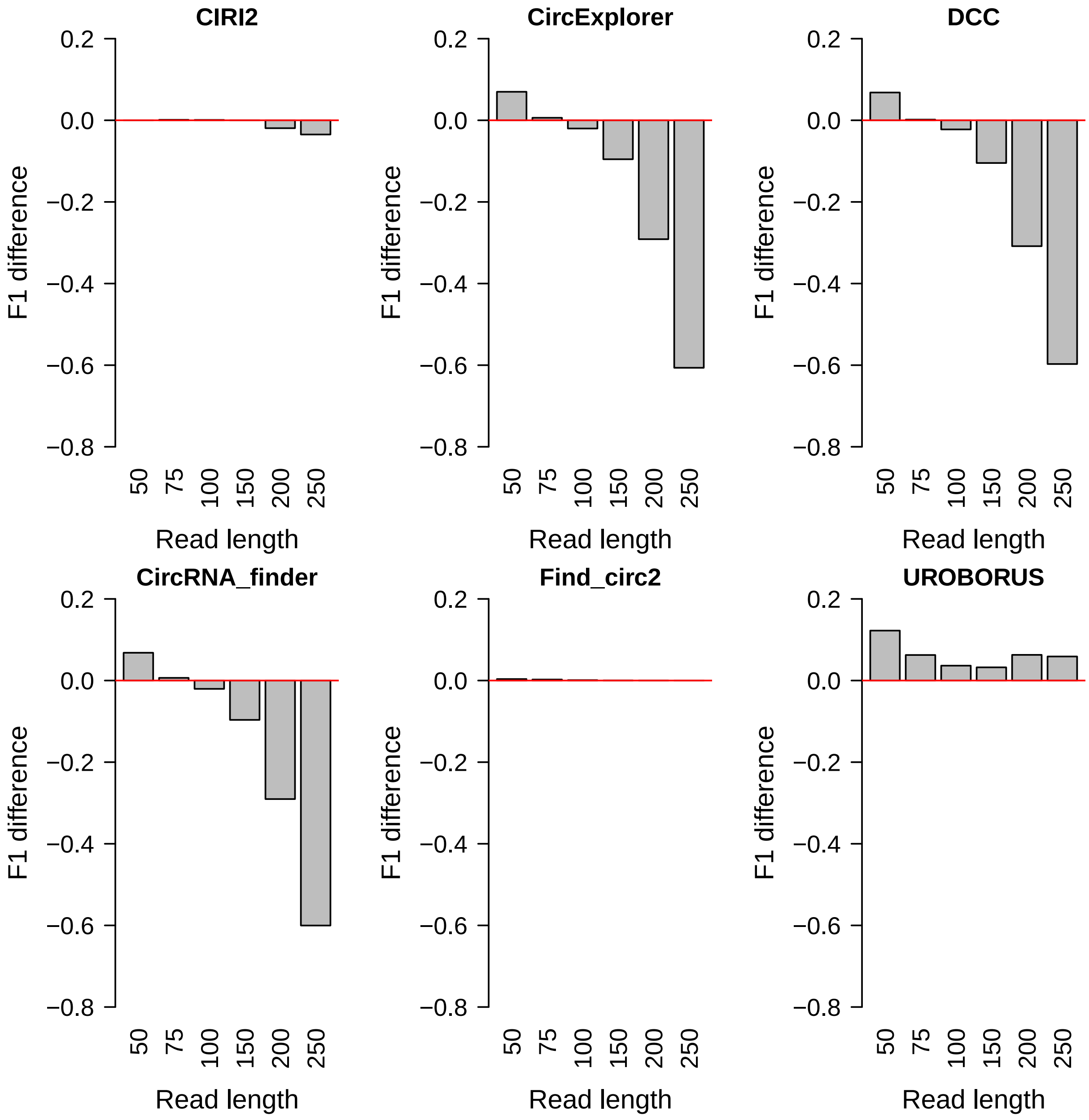

Figure S13: **The difference of F1 score between the results of the PE and simulated SE datasets in the ratio setting of 5-95.** The x-axis presents the read length of the simulated samples. The y-axis shows the F1 difference. A positive (negative) difference indicates the F1 score from the PE dataset is greater (less) than that of the SE dataset.

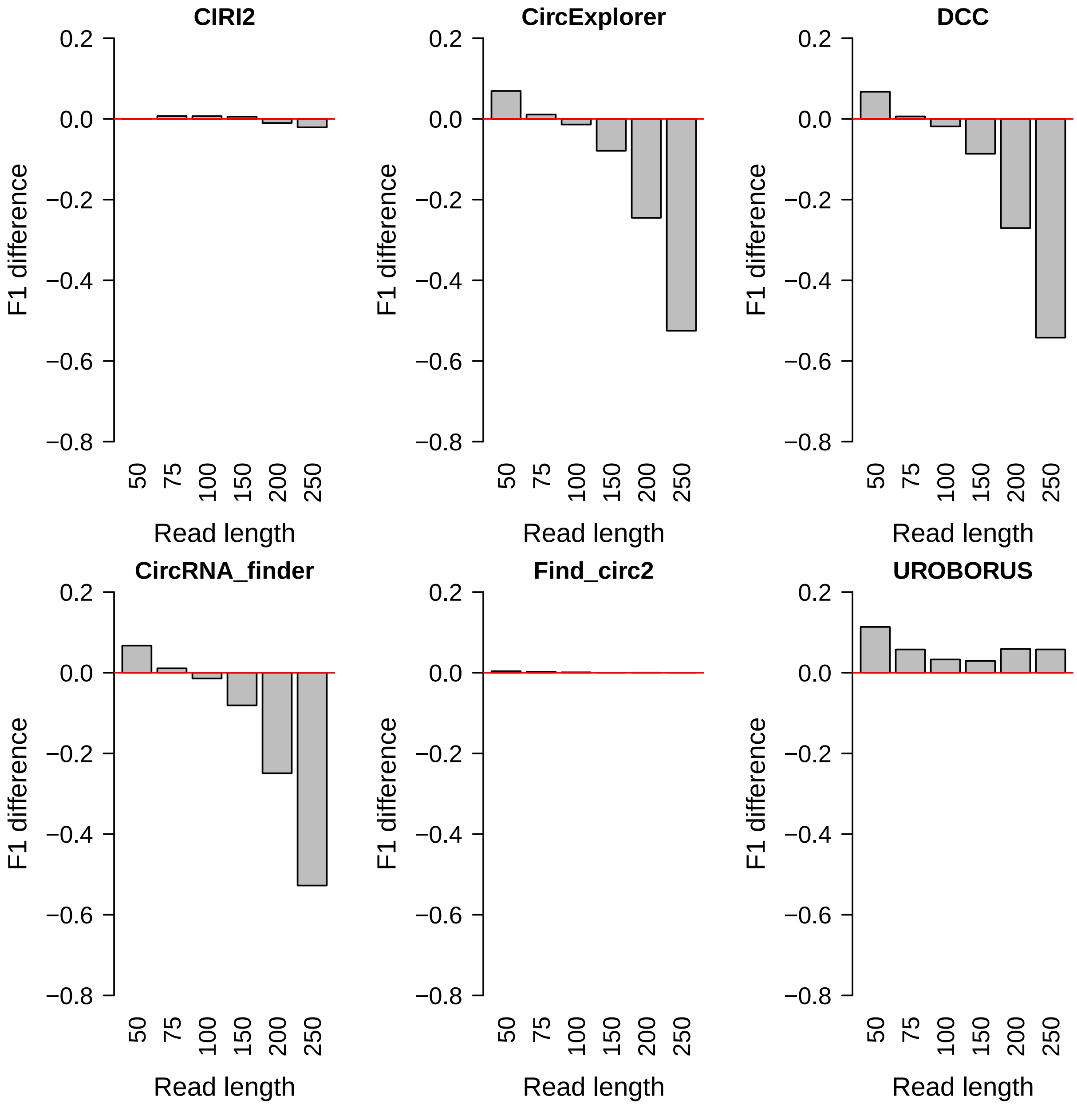

Figure S14: **The difference of F1 score between the results of the simulated PE and SE datasets in the ratio setting of 30-70.** The x-axis presents the read length of the simulated samples. The y-axis shows the F1 difference. A positive (negative) difference indicates the F1 score from the PE dataset is greater (less) than that of the SE dataset.

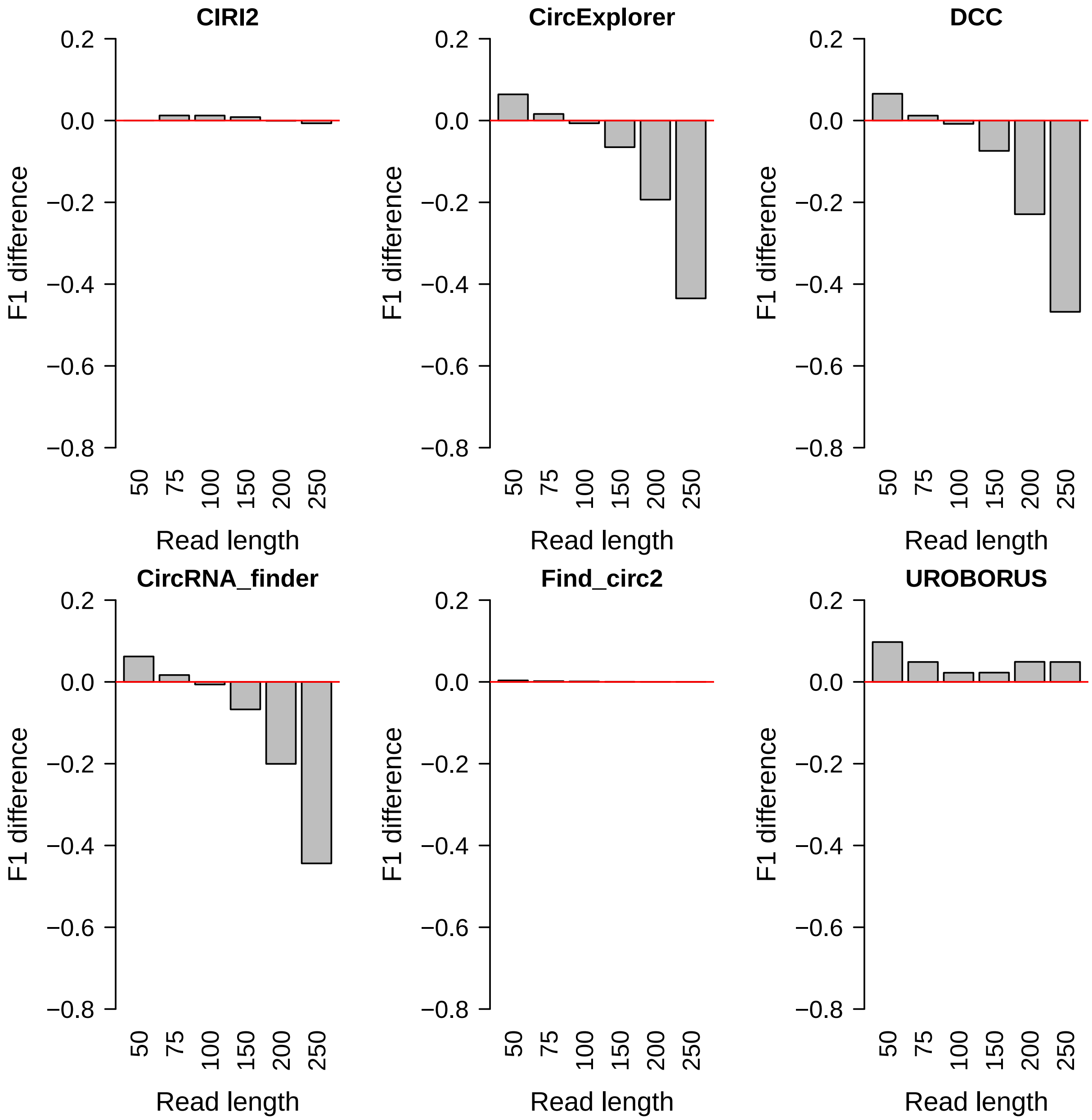

Figure S15: **The difference of F1 score between the results of the simulated PE and SE datasets in the ratio setting of 50-50.** The x-axis presents the read length of the simulated samples. The y-axis shows the F1 difference. A positive (negative) difference indicates the F1 score from the PE dataset is greater (less) than that of the SE dataset.

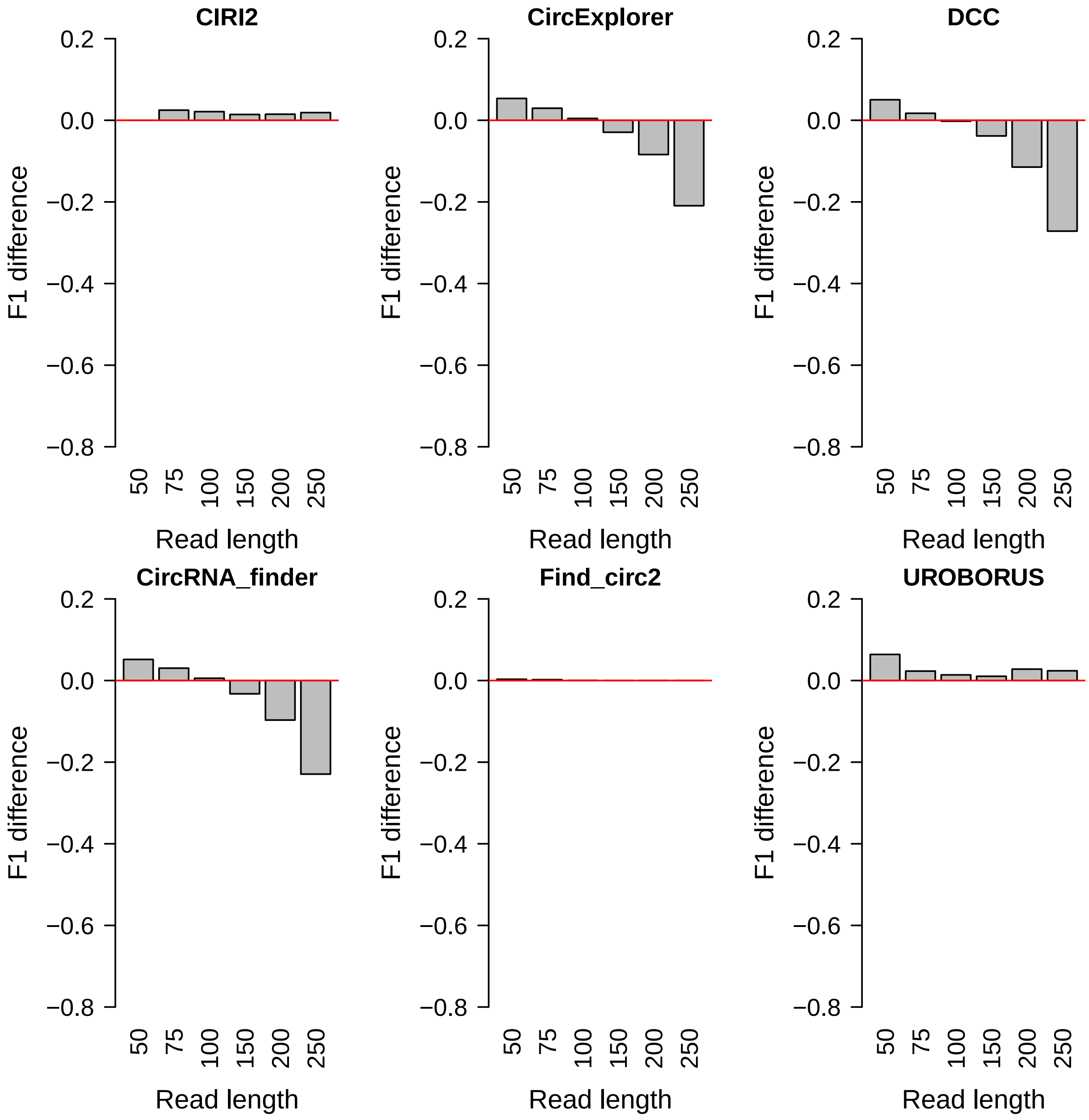

Figure S16: **The difference of F1 score between the results of the simulated PE and SE datasets in the ratio setting of 80-20.** The x-axis presents the read length of the simulated samples. The y-axis shows the F1 difference. A positive (negative) difference indicates the F1 score from the PE dataset is greater (less) than that of the SE dataset.

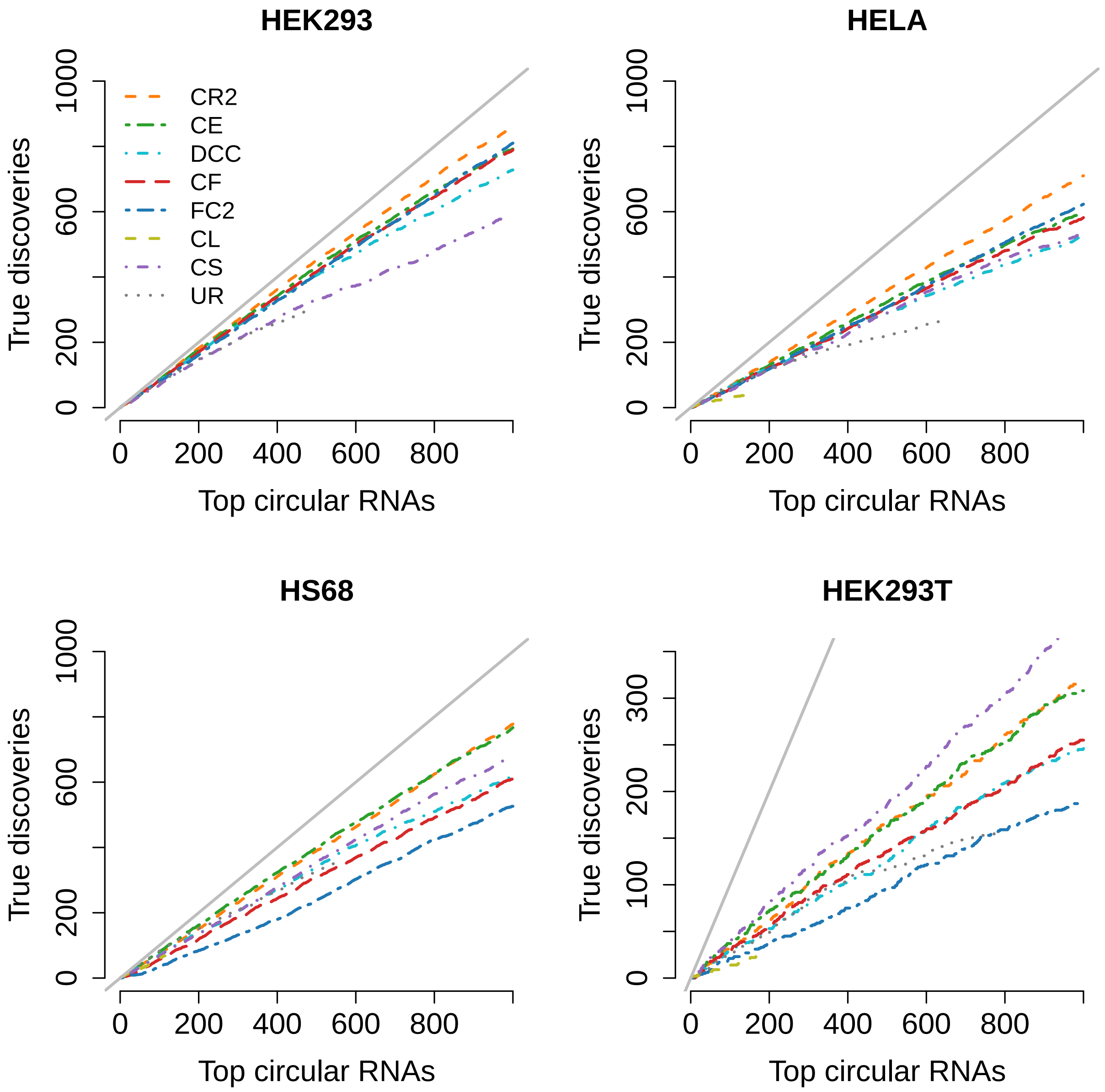

Figure S17: **Comparison of all circRNA detection methods in the single-end experimental RNA-Seq datasets using top 1000 circRNAs.** The x-axis presents the number of top circRNAs ranked by expression. The y-axis indicates the number of true positives (non-depleted circRNAs) in the top circRNAs. The black solid diagonal line presents the perfect true discovery rate. CR2: CIRI2, CE: CircExplorer, CF: CircRNA_finder, FC2: Find_circ2, CL:Clirc, CS: CircScan, UR: UROBORUS

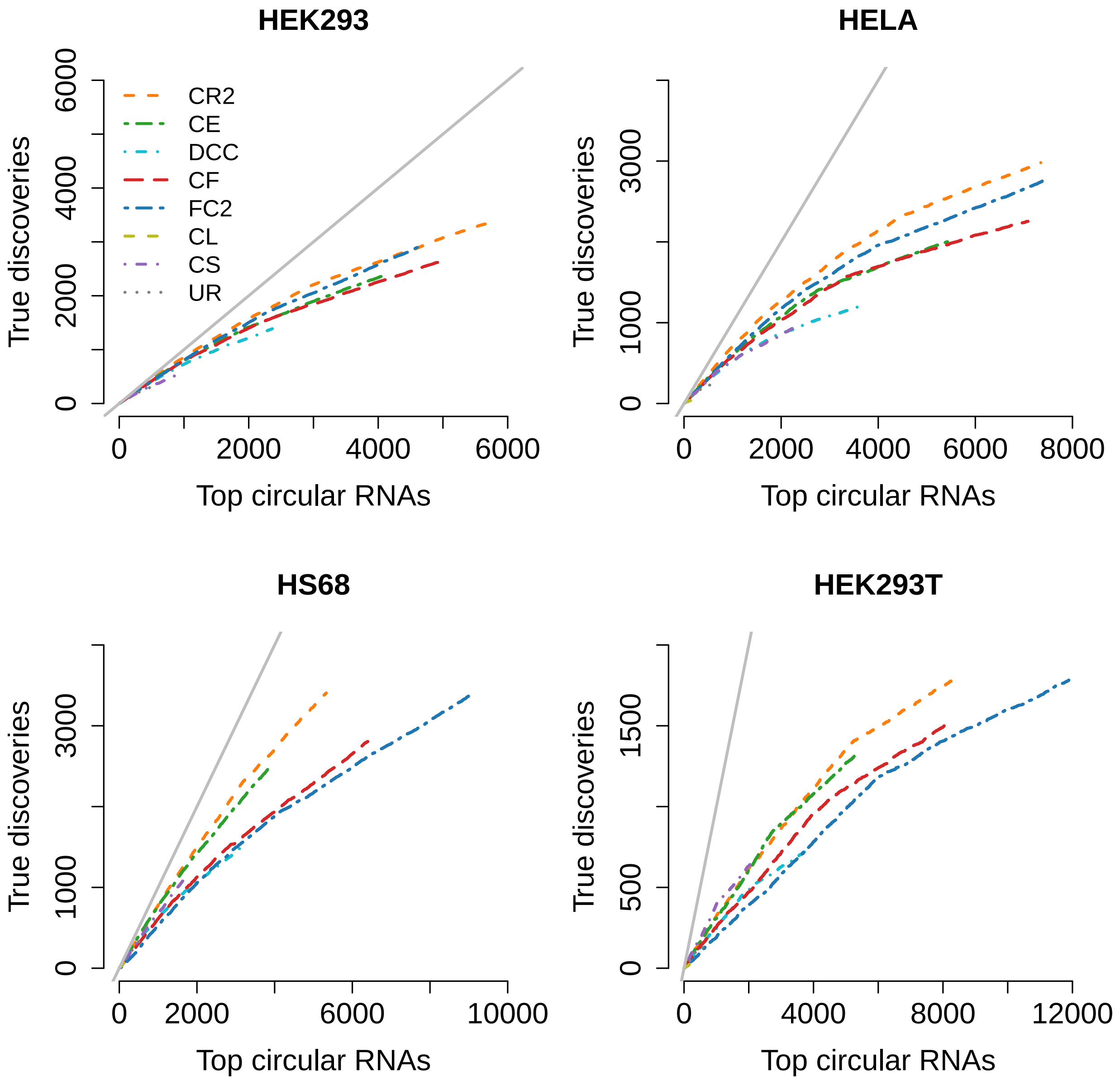

Figure S18: **Comparison of all circRNA detection methods in the single-end experimental RNA-Seq datasets using all circRNAs.** The x-axis presents the number of top circRNAs ranked by expression. The y-axis indicates the number of true positives (non-depleted circRNAs) in the top circRNAs. The black solid diagonal line presents the perfect true discovery rate. CR2: CIRI2, CE: CircExplorer, CF: CircRNA_finder, FC2: Find_circ2, CL:Clirc, CS: CircScan, UR: UROBORUS

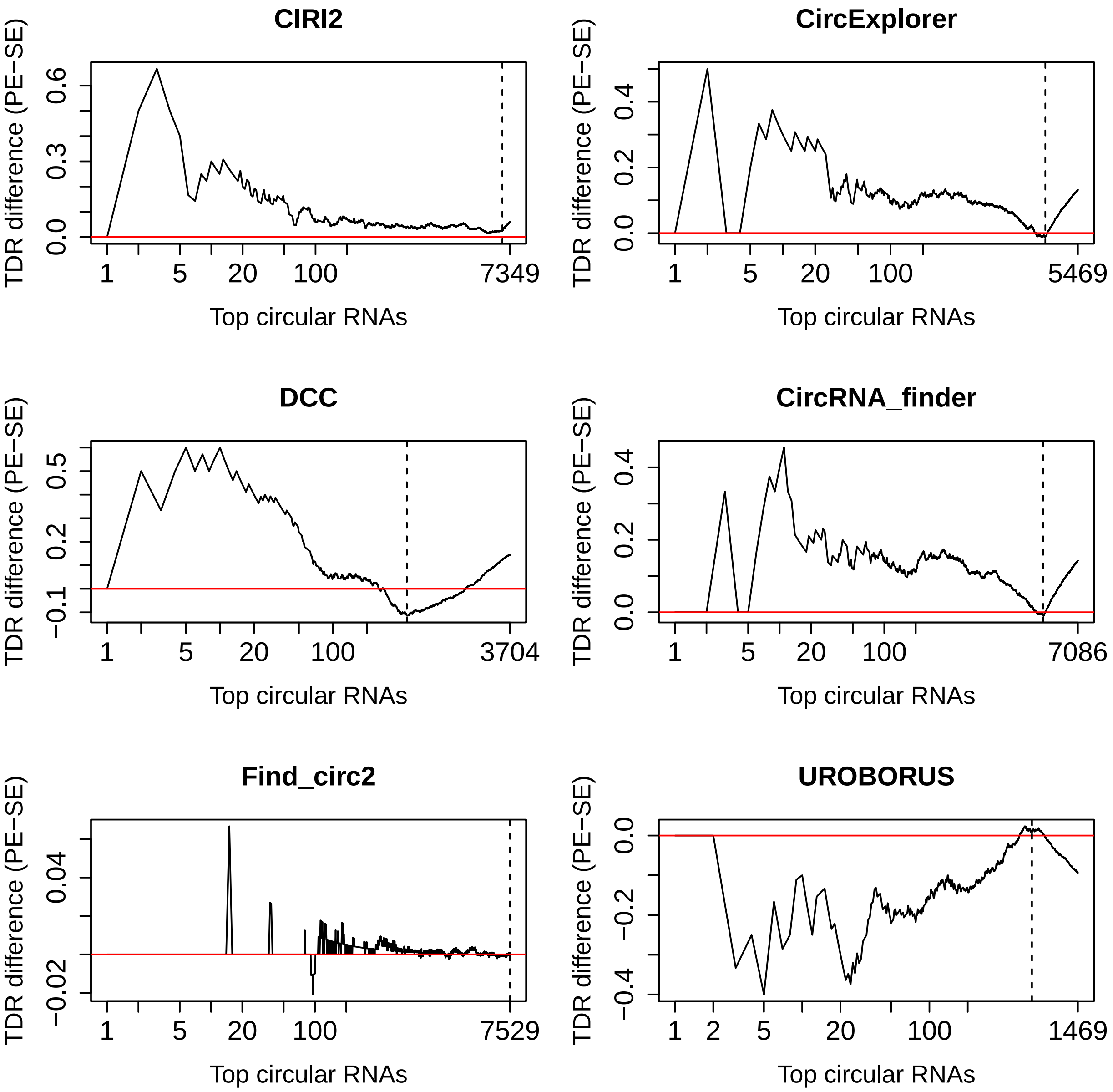

Figure S19: **Difference of true discovery rate between PE and SE data of HELA cell line across the RNA-seq based methods.** The x-axis presents the number of top circRNAs ranked by expression. The y-axis indicates the difference of true discovery rate (TDR) in the top circRNAs. If the TDR difference is positive which is above the red solid horizontal line, the TDR of the PE sample is greater than the TPR of the SE sample and vice versa. The black dash vertical line presents the number of circRNAs detected from the SE dataset for UROBORUS and the PE dataset for the other tools.

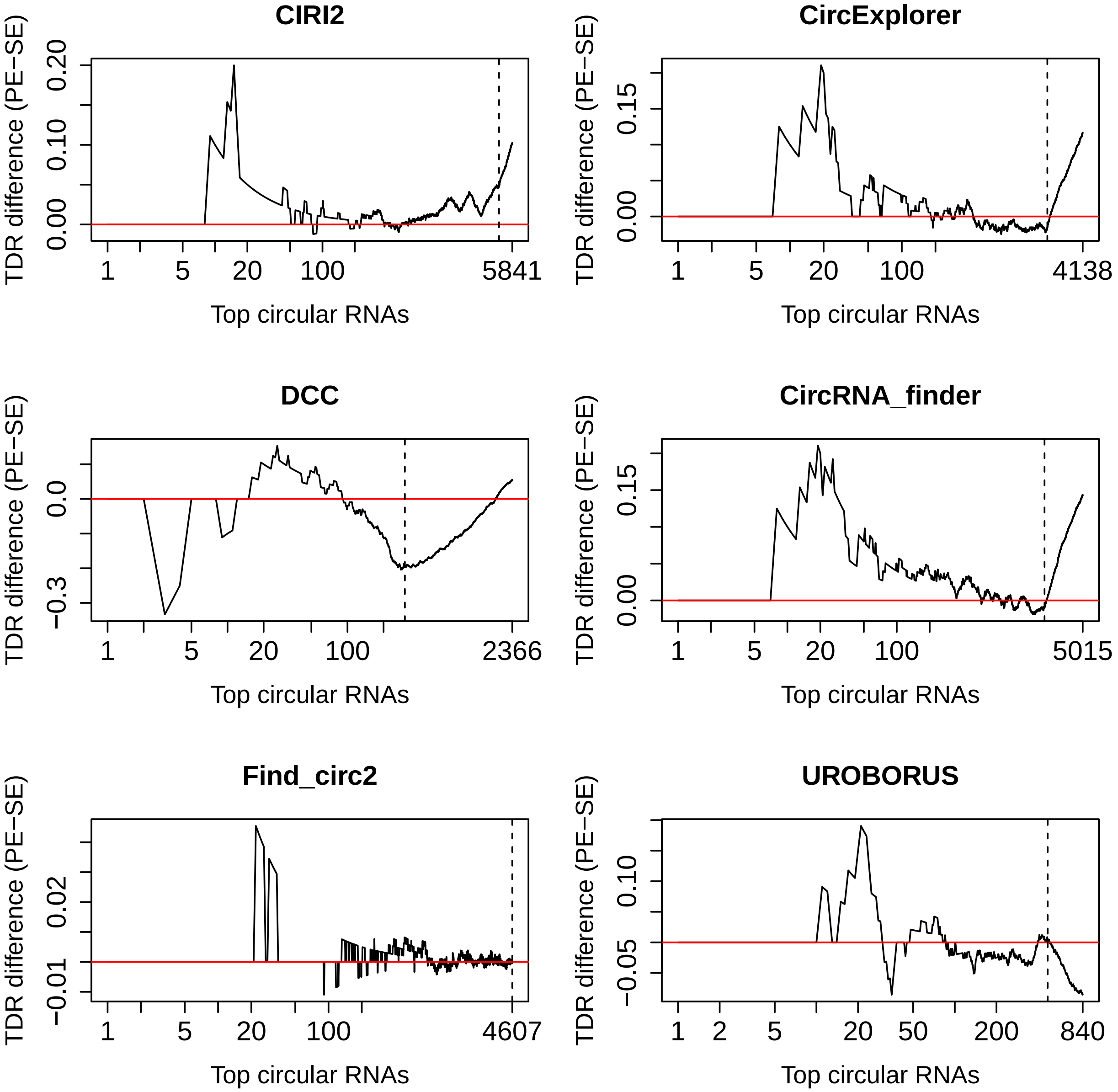

Figure S20: **Difference of true discovery rate between PE and SE data of HEK293 cell line across the RNA-seq based methods.** The annotation is similar as described in Figure S19

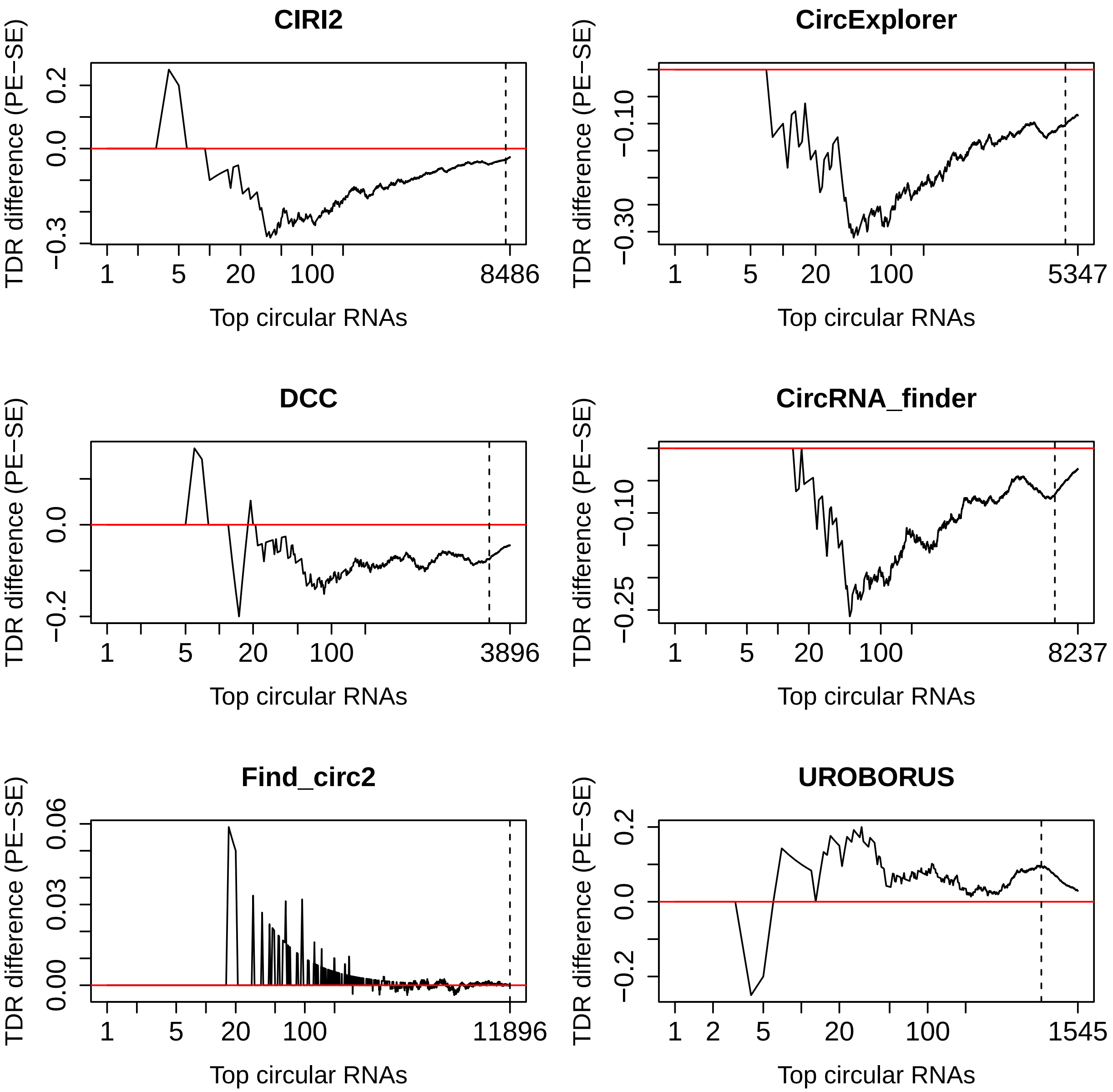

Figure S21: **Difference of true discovery rate between PE and SE data of HEK293T cell line across the RNA-seq based methods.** The annotation is similar as described in Figure S19

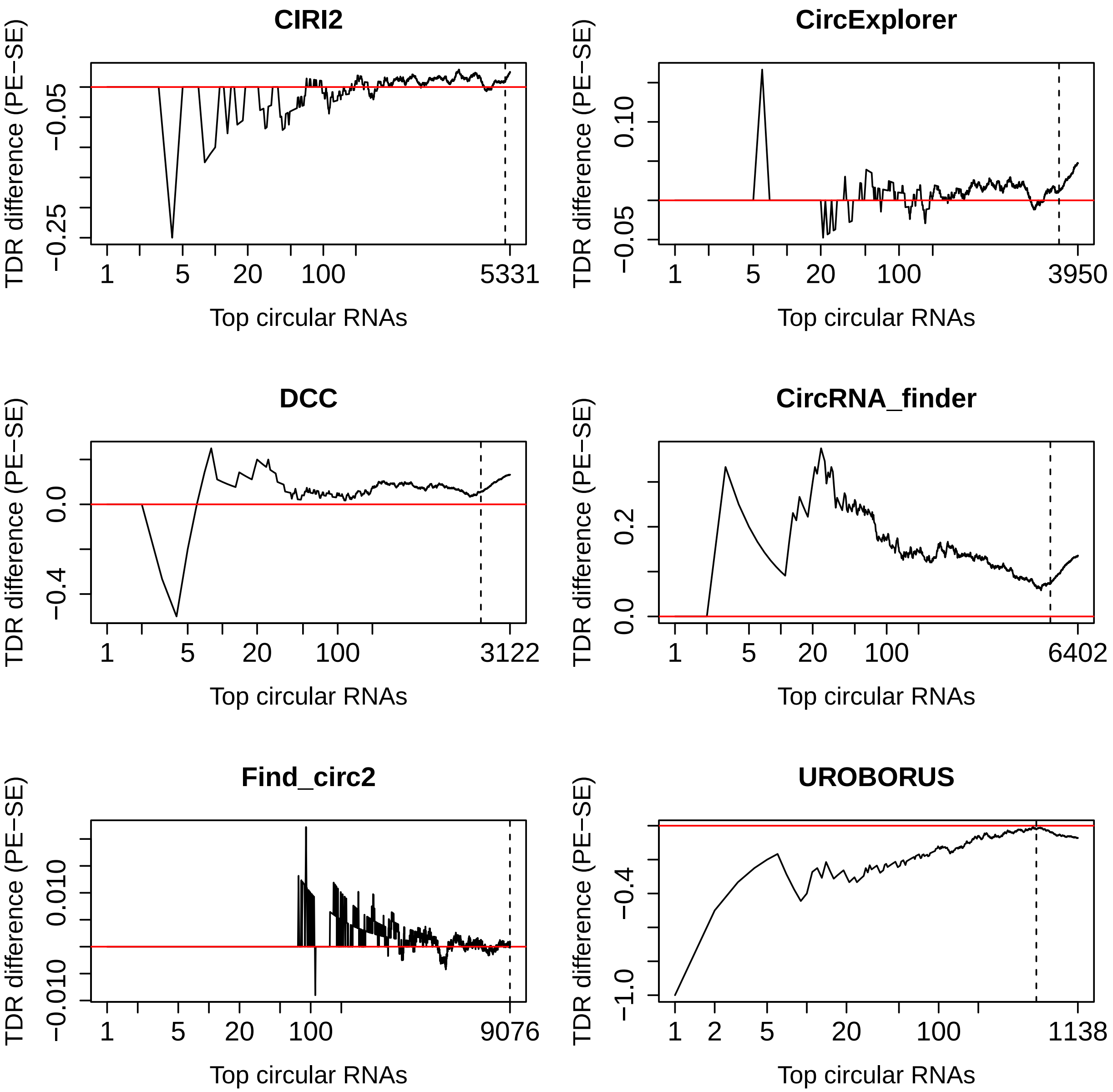

Figure S22: **Difference of true discovery rate between PE and SE data of HS68 cell line across th RNA-seq based methods.** The annotation is similar as described in Figure S19

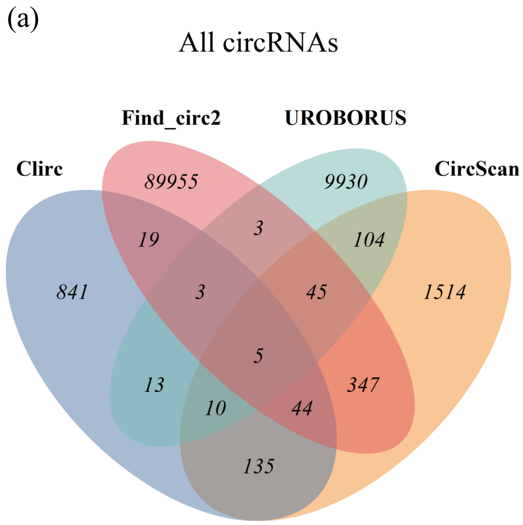

Figure S23: **CircRNAs discovered by CircScan, Clirc, UROBORUS and Find_circ2 in the CLIP-seq dataset**. (a) All identified circRNAs and (b) True positive circRNAs. The value in the parentheses presents the precision in each region.
